# Supplementary material for: Effectiveness of Bubble Continuous Positive Airway Pressure (BCPAP) for Treatment of Children Aged 1–59 Months with Severe Pneumonia and Hypoxemia in Ethiopia: A Pragmatic Cluster Randomized Controlled Clinical Trial
Source: J Clin Med. 2022 Aug 23;11(17):4934. doi: 10.3390/jcm11174934 (PMC9456562; doi:10.3390/jcm11174934)
Supplement: Supplementary file 1 [file jcm-11-04934-s001.zip › jcm-1817866-supplementary-Appendix SA2.pdf]

Appendix-SA2

Feasibility and Acceptability Followed by Effectiveness of Bubble Continuous Positive Airway Pressure (bCPAP) for Treatment of Children aged 1-59 months with Severe Pneumonia in Ethiopia: A *Pragmatic Cluster Randomized Controlled Clinical Trial*

Investigators:

1. **Dr Meseret Gebre (MD) -PI**
   Pediatrician
   ALERT Center
2. **Dr. Mohammod Jobayer Chisti (MBBS, MMed, PhD) -PI**
   Senior Scientist and Clinical Lead, ICU, Dhaka Hospital, icddr,b
3. **Professor Trevor Duke (MD, FRACP, FJFICM)- Co-PI**
   Paediatric Intensivist, RCH, The University of Melbourne
4. **Professor John D Clemens (MD, PhD)**
   Executive Director, icddr,b
5. **Professor Tahmeed Ahmed (MBBS, PhD)**
   Senior Scientist and Senior Director, NCSD, icddr,b
6. **Dr Abebe Genetu Bayih (PhD)**
   Director General, AHRI
7. **Rahel Argaw (MD)**
   Assistant Professor of Pediatrics and Child Health
   Pulmonology & Critical Care Fellow, Tikur Anbessa Specialized Hospital
8. **Meles Solomom (MSC)**
   Child Health Expert
   Maternal and child health directorate, Federal Ministry of health
9. **Asrat Demtse (MD)**
   Assistant Professor of Pediatrics and Child Health
   Neonatologist
10. **Bogale Worku (MD)**
    Professor of Pediatrics and Child Health
    Executive Director of the Ethiopian Pediatrics Society
11. **Abate Yeshidinber (MD)**
    Assistant Professor of Pediatrics and Child Health
    St. Paulos Milleneum Medical College
12. **Mulatu Birru (PHD)**
    Director, Knowledge Management Directorate
    Armauer Hansen Research Institute

January 3, 2022, Addis Ababa, Ethiopia

Table of Contents

Abbreviations 3

Operational Definitions 4

Summary 5

1. Background 8

    1.1 Burden of disease 8

    1.2 Management challenges 9

    1.3 Methods of Oxygen delivery 9

    1.4 Continuous Positive Airway Pressure (CPAP) 10

    1.5 Evidences supporting Use of Bubble CPAP 13

2. Rationale of the study 15

3. Research questions 16

4. Objectives 17

5. Methods 18

7. Work Plan 31

8. Budget ..... **Error! Bookmark not defined.**

Annexes 32

References 60

Abbreviations

BCPAP-bubble Continuous positive airway pressure

CPAP- Continuous positive airway pressure

CDP-Continuously applied distending pressure

FRC-Functional residual capacity

ICU-Intensive care unit

IMV-Intermittent mandatory ventilation

IPPV-Intermittent positive pressure ventilation

NPO_2-Oxygen initiated by nasal prongs

WHO-World Health Organization

Operational Definitions

- Severe Pneumonia: A child with cough or difficult breathing and any of the danger signs below [32]
- Hypoxemia (Oxygen saturation <90% or central cyanosis)
- Severe respiratory distress (grunting, very severe chest in-drawing)
- inability to breastfeed or drink, lethargy or reduced level of consciousness, convulsions

Treatment failure (11):

A. Presence of severe hypoxemia ($SpO2<85\%$) at any time after at least one hour of intervention plus severe respiratory distress when the child is receiving BCPAP/LF

OR,

B. If the patient developed the indication of mechanical ventilation when the child is receiving BCPAP/LF

OR,

C. If the patient died during hospitalization

OR,

D. If the patient left against medical advice (LAMA) due to lack of improvement or deterioration of the child during hospitalization

Summary

**Short Title**	Bubble CPAP for management of children with severe pneumonia
**Methodology**	Cluster randomized controlled clinical trial<br><br>Methodology:<br><br>1. **Stages I and II**: Feasibility/demonstration stage will be done as an internal pilot in 4 hospitals<br>Current treatment practice, facilities, and operational challenges will be evaluated for the introduction, clinical use and maintenance of bubble CPAP<br>2. **Stage III**: Implementation of bubble CPAP will be done in 12 hospitals<br>It will be done following a cluster randomized design<br>.<br><br>**Stages I and II**:<br><br>Primary outcome: operational challenges that may include availability of pulse oxymetry, IV cannula, IV antibiotics, oxygen supply system and nasal catheters for treating severe pneumonia<br><br>Secondary outcomes: prevalence of severe pneumonia associated hypoxemia, their treatment practices, adverse events, and mortality and treatment failure<br><br>The results of Stages I and II will be submitted to Food, Medicine and Health Care Administration and Control Authority (FMHACA) in Ethiopia for their formal review and subsequent approval for the commencement of Stage III.<br><br>**Stage III**:<br><br>Primary outcome: Treatment failure

	Secondary outcomes:<br><br>• Death<br>• Adverse events (pneumothorax, abdominal distension, nasal trauma, aspiration pneumonia)encountered.
**Research Sites**	*St. Paulos Millennium Medical College, Yekatit 12 and Tikur Anbessa Specialized hospitals, 14 district hospitals*
**Objectives**	Stages I and II<br><br>- To assess the feasibility and acceptability (not only by patients' care-givers but also by physicians and nurses) of bubble CPAP in treating childhood severe pneumonia in two tertiary hospitals in Stage I and in two district hospitals in Stage II<br><br>- To record adverse events following use of bubble CPAP in these settings<br><br>- To understand how much resource and time are needed to institutionalize and maintain bubble CPAP as a routine practice in the health system<br><br>Stage III:<br><br>- To determine therapeutic effectiveness of bubble CPAP compared to WHO standard low flow oxygen in reducing treatment failure in children admitted to hospitals with severe pneumonia and hypoxemia<br><br>- To determine therapeutic effectiveness of bubble CPAP compared to WHO standard low flow oxygen in reducing treatment failure & mortality in children aged 1-12 months admitted to hospitals with severe

	pneumonia and hypoxemia<br>- To record adverse events (pneumothorax, abdominal distension, nasal trauma, aspiration pneumonia) encountered.
**Number of Participants/Patients**	Stage I-30 children in each tertiary hospital<br>Stage II- 20 children in each general hospital (2 general hospitals)<br>Stage III-620 children in each arm of the study (6 hospitals in each arm)
**Main Inclusion Criteria**	- Age between 1 month and 59 months<br>-Meet WHO clinical criteria for severe pneumonia with hypoxemia<br>-Hypoxemia (Oxygen saturation <90% in room air)<br>-Parent/guardian gives informed consent to participate in the study
**Statistical Analysis**	A descriptive analysis of data of stages I and II will be performed to assess the level of feasibility and acceptability both at provider and patient levels<br>- In stage III we shall follow the principle of intention to treat (all children enrolled in the study will be included in the analysis irrespective of whether they have completed the study intervention). Primary and secondary outcomes will be analyzed using χ^2 or Fisher's exact tests as appropriate.

	-Continuous variables will be analyzed using the Student t-test or the Mann-Whitney test as appropriate.<br><br>-The primary outcome of the study is treatment failure and the secondary outcomes are death, pneumothorax, abdominal distention, nasal trauma, aspiration pneumonia.<br><br>-Primary and secondary outcomes will be compared by calculating relative risks (RRs) and their 95% confidence intervals.<br><br>- Log-linear binomial regression will be applied to adjust for covariates to evaluate the true impact of bubble CPAP in evaluating primary and secondary outcomes and to adjust for baseline differences.
**Proposed Start Date (including activities)**	1st July 2018
**Proposed End Date**	31th December 2022
**Study Duration**	54 months

1. Background

1.1 Burden of disease

Every year over 5.9 million children die globally, mostly from preventable or easily treatable diseases, and more than 95% of those deaths occur in developing countries (1). Pneumonia is the leading cause of death in children under 5 years of age, being responsible for at least 18% of all deaths in this age category [1]. Despite the provision of oxygen, antibiotics and supportive care, the case fatality rate for children with severe pneumonia and hypoxaemia is still high in health facilities of resource limited settings [2,3]. In Ethiopia, sixty-seven in every one thousand under-five children die before celebrating their 5th anniversary [1]. Acute respiratory infection, particularly pneumonia, being the leading cause of morbidity and mortality in the country [4] and accounts for 16% of all under-five deaths [1].

1.2 Management challenges

Despite its importance in virtually all types of acute severe illness, hypoxaemia is often not well recognized or managed in settings where resources are limited [5]. Oxygen treatment remains an inaccessible luxury for a large proportion of severely ill children admitted to hospitals in developing countries [5]. This is particularly true for patients in small district hospitals, where, even if some facility for delivering oxygen is available, supplies are often unreliable and the benefits of treatment may be diminished by poorly maintained and inappropriate equipment, poorly trained staff or inadequate guidelines [6].

There are many possible clinical options, depending on the availability of medical equipments in managing a child with severe pneumonia and hypoxia (6). In the developed world, in addition to antibiotics and supportive care, the use of high-flow oxygen therapy, humidified high-flow mixture of air and oxygen via a nasal oxygen cannula and continuous positive airway pressure via mechanical ventilator are possible options for treating children with moderate or severe respiratory distress in intensive care units (ICU) (6).

Mechanical ventilation is indicated for acute or chronic respiratory failure, which is defined as insufficient oxygenation, insufficient alveolar ventilation, or both. It can result from several conditions including pneumonitis, ARDS, cardiogenic pulmonary edema, laryngeal angioedema, acute severe asthma. Gullian Barre syndrome, flail chest, massive pleural effusion, pneumothorax, severe sepsis and septic shock [31].

1.3 Methods of Oxygen delivery

Oxygen can be delivered via nasal prongs, nasal or nasopharyngeal catheters connected to oxygen source from cylinder, concentrator or central piped oxygen [7]. Other less commonly used methods include use face-masks, head boxes, incubators and tents which are not recommended because they require high flow rates of oxygen and can be inefficient if oxygen supplies are limited.

While using nasal prongs, the maximum flow rate without humidification is 1 L/min in neonates, 2 L/min in infants, 4 L/min in preschool children and 6 L/min in schoolchildren. Higher flow rates without effective humidification may cause drying of nasal mucosa, with associated bleeding and airway obstruction. The maximum flow rate for nasal catheter should be set at 1–2 L/min for infants and older children because of the risk of high pressures directed into the airway (11).

An increasingly used method of respiratory support beyond standard flow oxygen therapy uses a humidified high-flow mixture of air and oxygen via a nasal oxygen cannula [8]. This method has also been used effectively for neonatal respiratory distress, acute viral bronchiolitis, and other disorders in developed countries. In non-randomized trials high-flow oxygen therapy has been associated with reduced need for CPAP in high-income countries, but there is no evidence of a reduced need for mechanical ventilation, and humidified high

flow nasal cannula oxygen therapy has not been widely available in resource limited settings in low and middle income countries [9].

1.4 Continuous Positive Airway Pressure (CPAP)

Continuous positive airway pressure (CPAP) is a continuously applied distending pressure (CDP) used for maintenance of an increased transpulmonary pressure during expiratory phase of respiration, in a spontaneously breathing patient. CPAP helps maintain the functional residual capacity (FRC) of infants by recruiting lost lung volume in children with severe pneumonia, reduces atelectasis, reduces ventilation-perfusion mismatch, improves oxygenation, and reduces fatigue in ventilatory muscles [10]. CPAP can be delivered using a conventional ventilator, bubble circuit or a CPAP driver with various types of interfaces like face mask, nasopharyngeal tube or nasal prongs [11].

CPAP is distinct from intermittent positive pressure ventilation (IPPV) or intermittent mandatory ventilation (IMV) in which inspiratory effort is supported by positive pressure, or breathing is taken over completely by the machine using positive pressure in both inspiratory as well as expiratory phases.

Bubble CPAP

Bubble CPAP is a form of CPAP oscillatory pressure delivery in which mechanical vibrations are transmitted to the chest secondary to non-uniform flow of gas bubbles across the downstream of a water seal and this system results in waveforms similar to those produced by high-frequency ventilation when recorded by a transducer attached to the infant's airway. The chest vibrations produced contribute to gas exchange by facilitated diffusion [11].

In its most basic form, pressurized oxygen from an oxygen cylinder is delivered to the nasopharynx of the baby. An underwater 'T tube' that acts as a blow off valve is interposed between the oxygen source and the baby. Adjusting the height of the water column above the exit of the 'T tube' can regulate the pressure in the system and the amount of CPAP delivered to the baby. The constant bubbling of gas through the blow off mechanism delivers the bubbling CPAP effect. Oxygen may be delivered by nasal prongs or more cheaply by a shortened endotracheal tube or a nasopharyngeal catheter (8F) inserted into the nose to a depth equal to the distance from the side of the nose to the front of the ear so that the tip of the catheter is just visible in the pharynx below the soft palate when the mouth of the infant is open [11].

Bubble CPAP has been used successfully in some referral hospitals in developing countries (10, 11, 12, 13). The circuit used in bubble CPAP oxygen therapy has three components [11]:

1. Continuous gas flow into the circuit: The gas flow rate required to generate CPAP is usually 5–10 L/min. This alone can generate CPAP, even without additional oxygen (FiO_2 = 0.21) but many neonates require supplemental oxygen. Therefore, the system also usually requires an oxygen blender, which connects an oxygen source (cylinder or concentrator) to the continuous airflow to increase the FiO_2.

2. A nasal interface connecting the infant's airway with the circuit (Fig. 1): short nasal prongs are generally used to deliver nasal CPAP. They must be carefully fitted to minimize leakage of air (otherwise, CPAP will not be achieved) and to reduce nasal trauma.

3. An expiratory limb with the distal end submerged in water to generate end-expiratory pressure: in bubble CPAP, the positive pressure is maintained by placing the far end of the expiratory tubing in water. The pressure is adjusted by altering the depth of the tube under the surface of the water.

Several commercial bubble CPAP machines are available (such as the system illustrated in Fig. 1). The price varies from several hundred US dollars to US$ 10 000.

Fig. 1. A bubble CPAP circuit connected to an infant by close-fitting nasal prongs

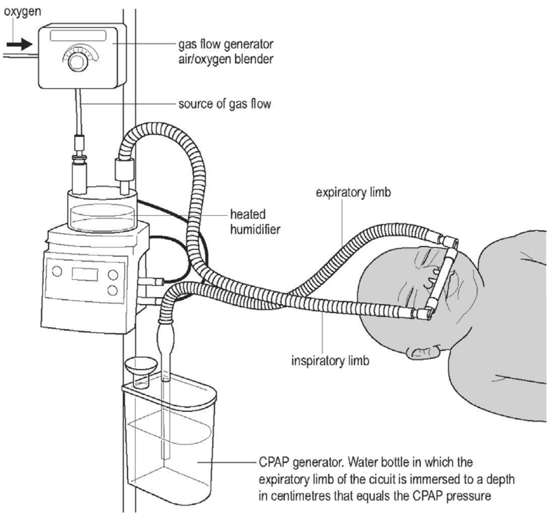

An inexpensive form of bubble CPAP can be made with standard nasal prongs. The method is shown in **Figs 2 & 3.** This system is used in several hospitals in Asia (e.g. Dhaka Hospital of icddr,b in Bangladesh) [10].
This locally made (Bangladesh) bubble CPAP will be reproduced in Ethiopia during the study period. The height of the bubble CPAP bottle (filled with water during its use for the

patient) is 17 cm, radius is 17 cm and diameter is 7.5 cm. Length of the tubing from the oxygen source up to the water filled plastic bottle ranges from 230 to 300 cm. A gas (oxygen) flow rate of 5–10 L/min is required for older children with pneumonia [10], while 3–4 L/min may be sufficient to generate CPAP in small neonates. In neonates born < 32 weeks' gestation, pure oxygen is not safe, as a high concentration can cause retinopathy of prematurity. Thus, another source of air flow, such as an air compressor or an oxygen blender, is required for premature infants. In older infants, who require a higher flow to generate CPAP, use of a 10 L/min oxygen concentrator is efficient.

Fig.2. An inexpensive bubble CPAP set up with modified nasal prongs [11].

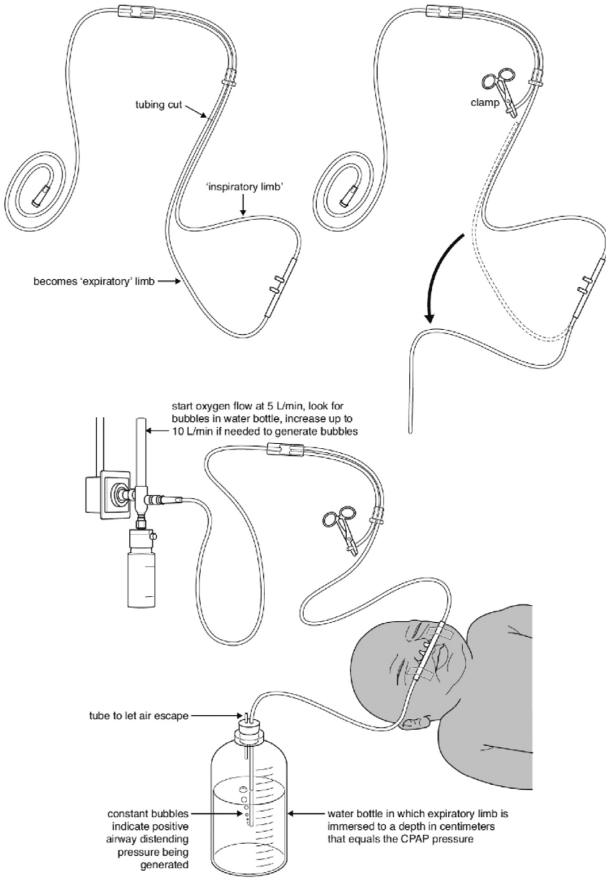

Fig. 3. Bubble CPAP with inexpensive modified nasal prongs can be run with an oxygen concentrator [11].

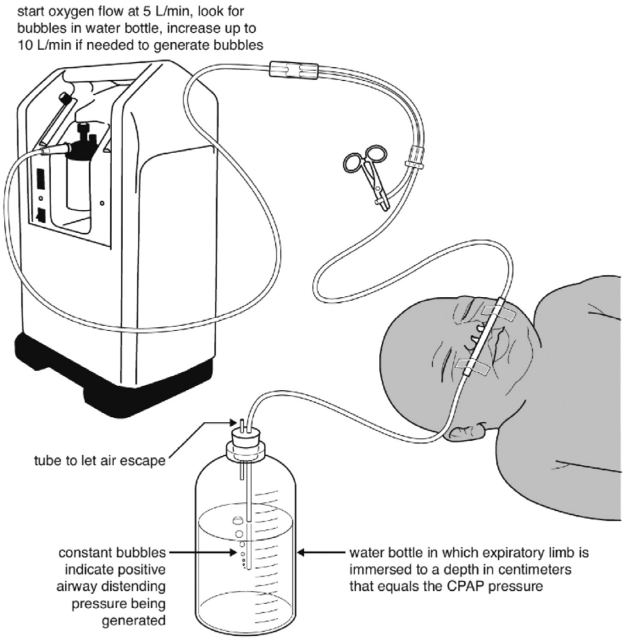

1.5 Literature Review

Most of the studies in evaluating the efficacy of bubble CPAP were done in newborns with acute respiratory distress syndrome both in developed and developing countries [11,12]. However, a recent systematic review identified 10 studies which included children with features of severe pneumonia [13]: four publications included data from developed countries such as two from USA [14,15], one each from United Kingdom [16] and Australia [17] while six other publications from developing countries such as three from India [18-20], one each from Fiji [21], Brazil [22], and 4 rural hospitals from Ghana (RCT) [23] focused on children beyond neonatal period.

All the ten studies were prospective and five among 0-28 days group [14,15,17,19,22] and one among >28 days group[23] were RCTs. These 10 studies evaluated the efficacy of BCPAP involving 3164 children: 3059 in children aged 0-28 days and 105 children >28 days old. The study methodologies, the outcomes recorded and results were heterogeneous, although severe respiratory distress (severe dyspnea, or grunting or severe chest retraction and/or hypoxemia) was the common component of all these prospective studies. Children treated with BCPAP therapy compared to other forms of oxygen therapy (head box oxygen, historical control, delayed BCPAP, or conventional oxygen therapy) in four of these studies (2 in children 0-28 days [17,21] and other in children >28 days [18,23]) had better outcomes ($p<0.05$). Primary outcomes were

comparable between BCPAP and ventilator driven CPAP in three RCTs in children 0-28 days old [15,19,22]. The primary outcomes of rest of the two studies treated with BCPAP oxygen therapy compared to low flow oxygen or variable flow nasal CPAP therapy in children aged 0-28 days were also comparable (p>0.05) [14,16].

On the basis of knowledge gap found from the systematic review in treating children with severe pneumonia and hypoxaemia with bubble CPAP especially in children beyond neonatal period, the Bangladesh trial was conducted which found a beneficial effect of bubble CPAP in these children. The trial done in Bangladesh showed that, among the children who received bubble CPAP for the treatment of severe pneumonia some children developed abdominal distension, and nosocomial infection, however, these adverse events were similarly observed with WHO standard low flow oxygen therapy in the same trial [11]. It is also important to note that bubble CPAP has been used routinely to support nearly 1,000 children with severe pneumonia and hypoxemia at the icddr,b hospital in Bangladesh with beneficiary outcome almost consistent with the trial outcome [24].

After the end of Bangladesh trial, an observational prospective study done in 77 Malawian children (1 week to 14 years) with progressive acute respiratory failure revealed that forty-one (53%) patients survived following bCPAP treatment, and an HIV-uninfected single-organ disease subgroup demonstrated bCPAP success in 14 of 17 (82%) [25].

Another prospective study, conducted in the Pediatric Emergency Unit of a teaching and referral hospital in North India which included 330 children (110 put on bubble CPAP) with clinical pneumonia and hypoxemia aged 1 month–12 years, they compared bubble CPAP with low flow oxygen and revealed that fifty-three children (25.9%) on low flow oxygen therapy were shifted to bCPAP for worsening distress and hypoxemia. There were a total of nine deaths (2.7%) in the study cohort; all deaths occurred in intubated children, of which only one child was initially in bCPAP group [26].

Another study done in 120 children under 2 years with moderate to severe lower respiratory tract infection revealed that bubble CPAP was successful in 72% of children [27].

A recent literature review which included 45 studies (17 clinical trials,11 literature reviews, 10 technical assessment, three reports of real world implementation in low resource settings, three cost analysis, one case report) also concluded that CPAP is a safe and effective method of treating infants and young children with life threatening respiratory distress and can be successfully adapted for use in resource limited settings [28].

A most recent meta-analysis involving eight RCTs that compared bubble CPAP with high flow nasal canulla and WHO standard low flow oxygen therapy revealed that bubble CPAP was more beneficial than high flow nasal cannula and low flow oxygen therapy in treating under-five children with serve pneumonia and hypoxemia [30].

2. Rationale of the study

The provision of mechanical ventilation for hypoxaemic children in developing countries through endotracheal intubation, is not available, feasible or affordable [11]. Nevertheless, a recent finding from a clinical trial in Bangladesh showed that oxygen therapy delivered by ultra low cost bubble CPAP is a possible alternative. According to Chisti et al., use of bubble CPAP significantly improved treatment failure and mortality outcomes in children with very severe pneumonia and hypoxaemia compared to those with standard low-flow oxygen therapy [10].

In countries like Ethiopia, where low-flow oxygen therapy is the only respiratory support for most of the children with severe pneumonia and hypoxaemia, having simple and effective methods of providing additional respiratory support could substantially reduce deaths from pneumonia. Bubble CPAP works by generating positive-end-expiratory pressure connecting the expiratory limb of a breathing circuit to a tube, which is submerged in water [11]. In the Bangladesh study, bubble CPAP, very simple to use and inexpensive, has been shown to be as effective as high-flow oxygen therapy but had better outcomes compared to WHO standard low flow oxygen therapy [10].

However, the feasibility and acceptability of bubble CPAP were not evaluated in childhood severe pneumonia in developing country set-ups, moreover, effectiveness of bubble CPAP has never been assessed for children with severe pneumonia in developing countries at a district hospital level where availability of ventilators and other respiratory support devices and high calibre professionals are scarce despite providing care for quite a number of children with severe pneumonia and hypoxemia. This implementation research comprising of a multicentre trial has been designed to have potential policy change for the management of childhood severe pneumonia and hypoxemia nationally and globally.

The use of traditional RCTs has a problem of speed, intense monitoring with high manpower and cost due to its efficacy nature and rarely produces findings that are directly put into practice.Recently concluded RCT in Bangladesh on bubble CPAP that was associated with significant reduction in mortality and treatment failure among Bangladeshi children having severe pneumonia and hypoxemia, was a single centre trial in an urban hospital, and the trial was conducted with intensive monitoring and additional study personnel.

Policy makers like WHO cannot change their policy until the Bangladeshi study results are reproducible in real life scenario of other country especially in government hospitals where manpower and monitoring are less, although a recent study with larger sample in Ghana supported the Bangladesh study [29]. It is therefore critical to study whether the study results are reproducible in other developing country settings. As the Ethiopian government health staff visited icddr,b Dhaka hospital and were impressed with the innovative low-cost approach of bubble CPAP in treating children with severe pneumonia and hypoxemia, they became interested to implement this low cost innovation in Ethiopian hospitals as resources are less there and treatment of hypoxemia in children with severe pneumonia is still done by standard low flow oxygen.

Cluster randomized controlled trials with implementation design, reasonably inform decisions about practice, address questions of major clinical and public health importance and produce results that can be generalized and applied in usual care settings. Because they produce real-world evidence, they help to close the translation gap between discovery and practice. However, as we are intending to perform this trial in hospitals where we will have the availability of pediatricians and will have dedicated corners for the study subjects, it will be a hybrid effectiveness trial with elements of both efficacy and effectiveness.

Therefore, we aim to investigate the feasibility and acceptability of bubble CPAP in two tertiary hospitals in Stage I and two district hospitals in Stage II, and effectiveness of bubble CPAP in Stage III with a cluster randomized trial for treatment of children with severe pneumonia. In Stage III the controls will be the children treated with the WHO standard low-flow oxygen therapy. Primary outcome will be death and secondary outcome will be treatment failure among children hospitalized with severe pneumonia in Ethiopia using a cluster randomized controlled clinical trial design of an implementation study. In all the hospitals where the studies in the different stages will be conducted, pediatricians and dedicated corners for the study subjects will be available.

3. Research questions

1. How feasible, acceptable, and safe is bubble CPAP in treating children having severe pneumonia as perceived not only by patients' care-givers but also by physicians and nurses in the initial two stages?
2. How effective, feasible, acceptable, and safe is bubble CPAP for the treatment of young children under five with severe pneumonia compared to the WHO standard low-flow oxygen therapy in reducing death (primary outcome) and treatment failure (secondary outcome) among hospitalized children in district hospitals in Ethiopia?

3. How much resource and time is needed to institutionalize and maintain bubble CPAP as a routine practice in the health system?
4. What are the main potential challenges in using bubble CPAP at different levels of health care delivery system?

4. Objectives

4.1 General Objective:

To evaluate the feasibility and acceptability of bubble CPAP in treating childhood severe pneumonia initially in two tertiary followed by two district hospitals (stages I and II) and finally to assess the effectiveness of bubble CPAP in reducing treatment failure in children with severe pneumonia in stage III compared with the WHO standard low-flow oxygen therapy among children hospitalized with severe pneumonia and hypoxemia in Ethiopia.

4.2 Specific Objectives

Primary Objectives

- To determine the effectiveness of bubble CPAP in reducing treatment failure in children admitted to hospital with severe pneumonia and hypoxemia compared to using WHO standard flow oxygen therapy up to the maximum flow that can run through nasal oxygen cannula in frontline district hospitals.
- To determine therapeutic effectiveness of bubble CPAP compared to WHO standard low flow oxygen in reducing treatment failure and mortality in children aged 1-12 months admitted to hospitals with severe pneumonia and hypoxemia

Secondary Objectives

- To assess the feasibility, acceptability, and safety of bubble CPAP in treating childhood severe pneumonia in two tertiary hospitals in Stage I, in two district hospitals in Stage II

- To examine the programmatic and behavioral acceptability of bubble CPAP at various health care levels (primary to tertiary) in Ethiopia in first two stages
- To identify and predict implementation level challenges and bottle necks of scaling up bubble CPAP to district level facilities
- To determine the effectiveness of bubble CPAP in reducing deaths in children admitted to frontline district hospitals with severe pneumonia and hypoxaemia compared to WHO standard flow oxygen therapy up to the maximum flow that can run through nasal oxygen cannula.

5. Methods

5.1 Study Stages:

Stage I. Testing feasibility, acceptability, and safety of locally constructed bubble CPAP in tertiary hospitals

Stage II. Investigation of feasibility, acceptability, and safety of bubble CPAP in frontline, district hospitals

Stage III. To evaluate the comparative therapeutic effectiveness of bubble CPAP compared to low flow oxygen in frontline, district hospitals

5.2 Detailed Description of Methods and Materials

Stage I. Testing feasibility, acceptability, and safety of locally constructed bubble CPAP in tertiary care hospitals

Study Design: During this Stage feasibility/demonstration stage will be done as a pilot in 3 hospitals where we will evaluate current treatment practice, facilities, and operational challenges for the introduction, clinical use and maintenance of bubble CPAP. Children with severe pneumonia and hypoxemia diagnosed by hospital physician will be enrolled consecutively and put on bubble CPAP. To have this enrolment among the eligible children, we will follow the WHO recommended “Management Algorithm of Children 1-59 months with Acute Respiratory Infections” (Annex 14)

Study setting: The study will be conducted at Tikur Anbessa, St. Paul's Millennium and Yekatit 12 Medical College hospitals. The hospitals are located in Addis Ababa, Ethiopia. All the hospitals are tertiary teaching hospitals. An average of 80-100 under five children with severe pneumonia are admitted to St. Paul's Millennium Medical College Hospital & Yekatit 12 hospital and 30-40 patients in Tikur Anbessa Specialized Hospital each month. The current practice of oxygen delivery methods in both hospitals is via low flow oxygen therapy and occasional use of locally constructed bubble CPAP for selected patients based on clinicians' decision. Children with severe pneumonia are also placed on IV antibiotics and other supportive care including maintenance fluid. Children will be placed on mechanical ventilators if they have any specific indication. The indications for mechanical ventilation are acute or chronic respiratory failure, which is defined as insufficient oxygenation, insufficient alveolar ventilation, or both. It can result from several conditions such as pneumonitis (infectious, aspiration), ARDS, cardiogenic pulmonary edema, upper airway obstruction (croup, laryngeal angioedema), lower airway obstruction (severe acute asthma), hypoventilation (drug overdose, Gullian Barre syndrome), severe sepsis and septic shock (25).

Inclusion criteria

- Age between 1 month and 59 months,
    - Meet WHO clinical criteria for severe pneumonia (these include a child with cough or difficulty in breathing and any of the danger signs below
    - Hypoxemia (oxygen saturation <90% or central cyanosis)
    - Grunting
    - inability to breastfeed or drink, lethargy or reduced level of consciousness, convulsions)
- Hypoxemia (oxygen saturation <90% in room air) is a must criteria
- Parent/guardian gives informed consent to participate in the study

Exclusion criteria

- Known congenital heart disease, asthma, or upper-airway obstruction
- Tracheostomy

- Pneumothorax
- Needs mechanical ventilation for any specific reason as decided by the clinician

Primary outcome

The primary outcome of this Stage of the study is the feasibility and acceptability of using bubble CPAP both at the patient level and at the health professional level in tertiary care hospitals

Sample and sampling procedure

This is a pre-implementation study aiming to test acceptability and feasibility of using bCPAP. So, we will use a convenient sampling technique. Thirty eligible children will be enrolled at this stage of the study from each site. All children who fulfill the inclusion criteria and whose parent/guardian give consent will be enrolled consecutively until the required sample size is reached.

Stage II. Testing feasibility, acceptability, and safety of locally constructed bubble CPAP in frontline, district hospitals

Study Design: During this stage children with severe pneumonia and hypoxemia will be enrolled consecutively and placed on bubble CPAP.

Study setting: This stage of the study will be done in two district hospitals to assess the applicability of bubble CPAP in less intensive facilities in the context of district level hospitals (where there is less manpower and equipment). The standard of care in these hospitals regarding oxygen delivery is low flow oxygen therapy.

Inclusion & Exclusion criteria are the same as Stage I.

Sample and sampling procedure

This part of the study aims to collect data on operational challenges before the major implementation trial so a convenient sampling will be used. Twenty illegible children will be enrolled at this stage of the study at each site. All children who fulfill the inclusion criteria

and whose parent/guardian give consent will be enrolled consecutively until the required sample size is reached.

Primary outcome –The same as with Stage I.

Qualitative methods for Stages I and II:

Qualitative Assessment

As part of a mixed-method approach, in Stage I a qualitative evaluation will be used alongside quantitative assessment to explore potential early operational challenges and opportunities for the introduction, clinical use and maintenance of bubble CPAP in order to rolling out and implementation of this innovative therapy in other public hospitals in Stage III after addressing all the challenges found in Stages I and II.

This qualitative assessment will be implemented through a participatory approach by actively engaging all stakeholders including consumers or beneficiaries, hospital health workers, clinicians, pediatricians, researchers and policy makers. The proposed participatory qualitative method will recognize that people within communities are best placed to identify the operational challenges and facilitators of BCPAP. The aim of this integrated approach is to provide the flexibility to fill in gaps in the available information, to provide different perspectives on complex, contextual, and multi-dimensional phenomena.

Qualitative data collection

Data will be collected concurrently in different episodes of quantitative assessment in Stages I and II. We will conduct interviews with individuals (e.g., a combination of key informant interviews and in-depth interviews) and groups (e.g. focused group discussions). Moreover, observation technique may be considered if interviews do not provide sufficient insights. The rationale of conducting Key Informant Interviews, In-depth Interviews, Focus Group Discussion (FGD) and Observations are described below**.**

Key Informant Interview

The rationale of choosing key informant interview for this study is to understand the health system issues related to the implementation of BCPAP in hospital settings, which will help to explore enabling policy environment in order to reach the project's goals. In addition, the key informant interview will help to recognize potential target population for in-depth interviews and FGDs, identify issues that may need further investigation, clarify survey findings, and generating study recommendations. The key informant will be selected from different level including hospital administrators, pediatricians and other policy level

stakeholders who will be knowledgeable about the particular issue related to study subject and influential in decision-making. In each Stage (I & II), 8-10 key informants will be interviewed; however, actual number will be determined based on data saturation and availability of informants.

In-depth Interview

The in-depth interviews will be conducted with patients' caregivers (who will be responsible for caring of the child during hospitalization; sometimes also called patient's attendant), research staff and hospital staff (physicians, nurses - who will directly provide the treatment) to understand the operational challenges and opportunities from their real life experience of introduction of BCPAP treatment. Their perceptions, motivation and attitude are important as they will be closely involved in receiving and providing the treatment. This interview will also help to explore additional-specific issues that might be supplemented with new information and clarify complex survey results. Additionally, in-depth findings from this interview will be used to triangulate to strengthen the validity of other findings from other data collection techniques. In each Stage (I & II), 10-15 in-depth interview will be conducted and numbers of interviews will be distributed equally in each of the study sites, however, actual number will be determined based on data saturation against explored board themes.

Focus Group Discussion (FGD)

The FGD will help to understand the willingness, affordability issues, and acceptance and to generate study recommendations for the introduction of BCPAP perfectly in the public health facilities. The FGD will be conducted with patient's guardians who play an important role in decision making related to treatment of the child and hospital staff. In each Stage (I & II), 8 FGDs will be conducted; however, actual number will be determined based on data saturation.

Observation

Multiple observation sessions may be arranged at hospital settings, if interviews do not provide sufficient insights. The aim of this observation might help to explore more insights from real-life experiences for introduction of BCPAP, service provider, child and caregivers' responses to the treatment of BCPAP. For this, the study team may spend time in the ward for observing and talking (informal conversation) to the caregivers and service providers (health worker, nurses or physicians) when they will be closely involved in providing treatment and child caring respectively. More specifically the observation may be held to understand:

- Ward environment (e.g., availability of bed in pediatric ward, attitude of physicians/consultants, nurses, health workers towards patients and their caregivers, cleanliness, electricity and other facilities, etc)
- Treatment practices (e.g., follow up visits, medication, interaction between patients' caregiver and hospital staff, patients' response to BCPAP, availability of oxygen support, the process of preparing the BCPAP circuit and functioning (capture if there is any difficulty, etc.)
- Treatment outcomes (e.g., well and discharged; diagnosed as a chronic illness and discharged with medication; died at the hospital).

Interviews, FGDs and observation will be conducted using flexible semi-structured guidelines (see annex-11) and equal in numbers in each of the study sites. As is the nature of qualitative study, guidelines might be revised for Stage III data collection based on experience and study findings of Stages I and II. Study participants will be selected purposively based on the characteristics of patients and caregivers, experience and knowledge of participants about the study subject. The data collection team will be males and females with a master's degree in Anthropology or Sociology as well as few years experience in collecting qualitative data. They will be trained by qualified Anthropologist.

For qualitative assessments, following number of participants will be interviewed for primary data collection in different episodes of quantitative assessment:

**Qualitative Assessment**	**Data collection techniques**	**Number of participants**
	Key Informant Interview	8-10
	In-depth Interview	10-15
	Focus Group Discussion	20-30
	Observation	
	**Total participants**	38-55

Data Analysis: Descriptive analysis of level of feasibility and acceptability will be performed by calculating the proportion of challenges such as lack of availability of oxygen, intravenous cannula, lack of assessment of vital sign etc.

For qualitative part: According to the nature of qualitative assessment, analysis will begin with the first field activities (in-depth interview, observation and FGD) and lead to improvement as the study proceeds. Field notes and dairy will be reviewed by the research team on a regular basis (initially, daily). For analyzing data, the processes will follow a sequence of inter-related steps that includes reading, coding, displaying, reducing, and interpreting. At first, transcripts will be carefully read, and then coding of data will begin. Reading and coding will initiate while data are being collected. The data-display and reduction process will be conducted once all data have been collected. Even during data display and reduction, the investigators will loop back through earlier steps to refine codes, re-read texts, and revise some aspects of analysis. After reading, re-reading, and coding the text, the main themes will be finalized. However, the complex issues will be presented in some verbatim forms. Each theme will then be examined separately and fully within the available data.

Stage III. Testing effectiveness of locally constructed bubble CPAP in general hospitals.

Study Design: During this Stage, a two arm cluster randomized clinical implementation trial will be undertaken at six district hospitals per arm. The study arms are:

i) Intervention arm (young children with severe pneumonia and hypoxemia treated by bubble CPAP) and

ii) Control arm (children treated with the WHO standard low-flow oxygen therapy up to the maximum flow rates for nasal cannula therapy without humidification)

Study setting

The study will be done in 12 district hospitals (6 in each arm) from all regions throughout Ethiopia. District hospitals will be selected randomly from all regions throughout the country. (Annex 13)

Inclusion criteria & Exclusion criteria - Same as Stage I

Primary outcome

The primary outcome of the study is treatment failure. According to this protocol treatment failure will be declared if any of the following criteria is met:

B. Presence of severe hypoxemia (SpO_2<85%) at any time after at least one hour of intervention plus respiratory distress when the child is receiving BCPAP/LF

OR,

C. If the patient developed the indication of mechanical ventilation when the child is receiving BCPAP/LF

OR,

D. If the patient died during hospitalization or within 72 hours of left against medical advice (LAMA)

OR,

E. If the patient left against medical advice (LAMA) when the patient is still on study intervention

Secondary Outcomes

- Death during hospitalization
- Deaths during hospitalization plus within 72 hours of LAMA
- Pneumothorax
- Aspiration pneumonia
- Length of hospital stay
- Incidence of nasal trauma, gastric distention, shock and air leaks
- Duration of bCPAP

Sample and sampling procedure:

In this trial, our recruitment goals are approximately a total of 1240 children (620 children each for LF oxygen and bubble CPAP therapy) for a power of 80% and effect size of 50% (reduction of treatment failure, primary outcome of the study, from chart analysis 10% treatment failure in 2019 in comparison facilities) for 12 clusters (103-104 patients in each cluster), adjusted for a intra-cluster correlation coefficient (ICC) level of 0.003 required for cluster randomization. For the calculation of sample size we have used following formula:

Where, DF, Design effect=1+δ(m-1) [here DF=1.52],

n_1= Required minimum sample size per arm, δ= the inter cluster correlation, m= the number of patients in each cluster [103.3 in this study],

P_1= the percentage of treatment failure of children with pneumonia receiving standard treatment (low flow) = 10%,

P_2= the expected percentage of treatment failure of children with pneumonia who will receive intervention (bubble CPAP) = 5% [we have observed 5% treatment failure in two district hospitals during the stage II of our study which correspond 50% effect size], Z_α= the z-score value [here, Z_α =1.96 at 5% level of significance], Z_β= the z-score value [here, Z_β =0.84 at 80% power]. We are intending to enroll same number of children (103-104) having our eligibility criteria from each hospital (cluster) irrespective of receiving intervention (bubble CPAP) or standard treatment (low flow).
As a run-in period we will enroll 2 participants in each hospital which will be excluded from final analysis.

Data collection: Data will be collected by individual patient assessment through systematic observations using a structured questionnaire. Socio-demographic and different clinical data will be collected. The questionnaire will include the baseline characteristics, intervention and outcome data. All study data will be collected by dedicated study personal at sites.

Data quality assurance

The study site supervisors will follow the randomization process and make sure that appropriate clinical follow up is done for the study participants and data collection tools are filled appropriately.

Supervision visit will be made to each study site every quarter by the principal investigator and the co-investigators to randomly assess the completeness of data. Moreover, by

random visit the trueness of data collection would be evaluated. Furthermore, data and safety monitoring board (DSMB) constituted by the IRB may randomly visit the study site and check any data to evaluate the integrity of data. The DSMB will also sit at least once after every 6 months to know the study progress and data procedure.

Study setting

The study will be done in the 12 district hospitals (6 in each arm) which are part of the effectiveness study.

Data analysis: Quantitative data will be analyzed using STATA -14 following the principle of intention to treat. Treatment failure and/or death will be analyzed using χ^2 or Fisher's exact tests. Primary and secondary outcomes will be compared by calculating relative risks (RRs) at 95% confidence interval. Log-linear binomial regression will be applied to identify the predictors of primary and secondary outcomes after adjusting for potential confounders. Continuous variables will be analyzed using the Mann-Whitney test.

5.3 Study procedures

5.3.1 Setting up the research team

There will be 17 research teams, one for each study site. The research team will consist of two nurses, one general practitioner and one pediatrician. There will be a central technical team composed of pediatricians, neonatologists, child health program experts and research experts. One pediatrician in each study hospital will be ensured and responsible for the monitoring of study activities.

Fig.4. Structure and responsibility of the research team

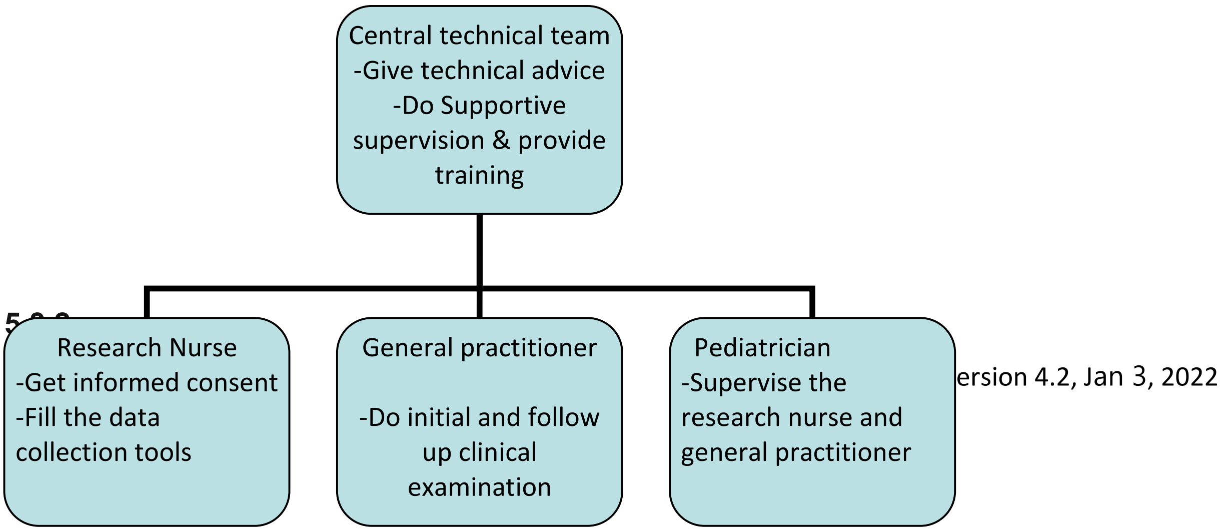

5.3.3

5.3.4 Training

For the feasibility & effectiveness study a two days training will be given to two nurses, one general practitioner and one pediatrician per facility. The training will be on how to explain the information sheet and get informed consent from parents/caregivers of study participants. They will also be trained on the data collection tools and preparation of locally constructed bubble CPAP. The trainers will be from the central technical team.

For the acceptability study a one day training will be given to qualitative data collectors on facilitating focused group discussion and conducting in depth interviews.

6. Ethical consideration

The study protocol will be submitted to Tikur Anbessa Specialized Hospital, St. Pauls' Milleneum Medical College, AHRI Institutional Ethical Review Board and National Ethics Review Board of Ethiopia and icddr, b IRB for ethical approval. Written informed consent will be obtained from parent or guardian of each study participants.

All data from the study participants will be kept in a locked cabinet at AHRI and will only be accessed by the study investigators. During publication, all patient identifiers will be removed in order to keep confidentiality.

Data Safety monitoring board (DSMB) will be established which will consist of one Pediatrician, one statistician, one Epidemiologist and Pediatric Intensivist who have knowledge on bubble CPAP. Results will be reported to the DSMB every 3 months and interim analysis will be done at 6 months of Stage III trial after which actions will be taken accordingly. All severe adverse events and deaths will be communicated to the DSMB within 24 hours.

7. Project Risks and Risk-Management strategies

This study does not introduce major risks to participants beyond what they would normally face in their condition. As it will be an interventional study with bubble CPAP oxygen therapy, side effects such as nasal trauma, nasal obstruction, gastric distension and pneumothorax are occasionally reported while using bubble CPAP in neonates but such complications are extremely rare in older children. In order to minimize those risks, all the necessary precautions will be taken with routine follow ups. We shall not perform any laboratory investigations for the study purpose except those required for routine care as determined by the hospital clinicians. Breaching medical confidentiality is also a risk. Training and standard SOPs will be implemented to help avoid these problems.

If a significant event caused as a direct result of participation in the study, the study staff will document the incident, inform the site PI, and local medical costs incurred, will be covered by the study's local medical malpractice insurance that is provided by the study.

8. Declaration of potential conflicts of interests

We declare no competing interests.

9. Recording and reporting of adverse and serious adverse events:

Following standard operating procedure, the study staff will document all incidents of adverse events and serious adverse events that are associated with participation in the study, inform the site PI, and local medical costs incurred will be covered by the study's local medical malpractice insurance that is provided by the study. Any serious adverse event (whether it is related to the intervention or not) and the actions taken will be reported to FMHACA within 48 hours of occurrence. In addition, summary of adverse events will be reported to FMHACA quarterly.

Indemnification and insurance:

The insurance policy will include both classical clinical trial insurance and indemnification insurance. A local insurance company will be responsible for them. The title of the trial, amount of insurance in respect of each participant, the number and/ or name of health facilities involved in the study are indicated in the insurance certificate.

Protocol deviation

In case of protocol deviations, a deviation form will be filled out by study staff and sent to the DSMB within 72 hours of the events.

10. Amendment of the protocol

If we need any amendment of the protocol during the study period we will provide amendment request to IRBs at AHRI, NREREC, and icddr,b.

11. Termination of the trial will be decided by the data safety monitoring board after analyzing quarterly report of the trial, and before termination the study sponsor and PI will inform IRBs at AHRI, NREREC, and icddr,b and FMHACA.

12. Final reporting, dissemination and publication

After the end of the study the results will be reported nationally and internationally by attending national and international scientific conferences and by arranging dissemination programs in Ethiopia, Bangladesh and other developing countries. Additionally we will publish the findings in international peer reviewed journal(s). Both AHRI and icddr,b will be the owner of the collected data. However, all investigators will have access to the data and those fulfilling international authorship criteria will be included as authors in manuscripts reporting the study results. For the main manuscript, the study PIs (Drs Meseret and Chisti) of this study will be the co-first authors of the main manuscript. One investigator from the Ethiopia and one from icddr,b (Professor J Clemens) will be the co-senior authors of this manuscript written from this study. If there will be more than one manuscript from this study, first authorship will be discussed and allocated according to international standards of authorship before preparing the draft.

Roles of icddr,b, AHRI, and FMOH

a. Roles of icddr,b:

i) Conceptualize and design of the study in collaboration with AHRI
ii) Attaining the funding for the study from SIDA Sweden for the collaboration
iii) Assist in procuring equipment for the study following signed memorandum of understanding between icddr,b and AHRI.
iv) Externally supervise & monitor the study following SOP
v) Provide training for staff development following SOP
vi) Provide technical and logistical support to the study, including on-site support following signed memorandum of understanding between icddr,b and AHRI.
vii) Assist in developing data management system for data entry, cleaning, analysis and reporting of the research findings following SOP.
viii) Help local PI to organize training for study personnel from each research site following SOP

b. Roles of AHRI:

i) Conceptualization and design of the study in collaboration with icddr,b

ii) Attaining the funding for the study from DFID Ethiopia for the collaboration

iii) Recruit and train staff

iv) Supervise and monitor the study
v) Assist in procuring necessary supplies for the study
vi) Make the necessary payments for the study personnel
vii) Development of data management system in collaboration with icddr,b for data entry, cleaning, analysis and reporting of the research findings following SOP
viii) Organize training for study personnel from each research site

c) Roles of FMOH

i) Provide support letter to study sites for the conduct of the study

ii) Follow the general progress of the research project

12. Work Plan

Activities	1	2	3	4	5	6	7	8	9	10	11	12	13	14	15	16	17	18	19	20	21	22	23	24	25	26	27	28
Development of protocol	■																											
Clearances at icddr,b and AHRI		■	■	■																								
Training of trainers at icddr,b					■																							
Selection, randomization, and training of clinical sites: care, data collection						■																						
Trial in two tertiary hospitals (Stage I)							■	■																				
Trial in two rural district hospitals (Stage II)									■	■																		
Implementation Stage: main trial (Stage III)											■	■	■	■	■	■	■	■	■	■	■	■						
Final cleaning and freezing of data set																							■					
Analysis and report writing																								■	■	■		
Dissemination, policymaker discussions																											■	■

Annexes

Annex1. Information sheet for Parents/Guardians of children with Severe pneumonia (Stage I & II)

Title of the study*: Feasibility, acceptability, safety, and efficacy/effectiveness of bubble continuous positive airway pressure (CPAP) for treatment of children Aged 1-59 months with severe pneumonia in Ethiopia: a cluster randomized controlled clinical trial*

Purpose of the research

Pneumonia is a disease caused by infection in lungs. Pneumonia may become severe and require oxygen therapy along with other medication. There are different forms of delivering oxygen to a child with severe pneumonia. World Health Organization recommends low flow oxygen therapy for the children who develop severe form of pneumonia with the lack of oxygen in blood. This oxygen therapy is helpful to reduce pneumonia related deaths in children. In many hospitals in Ethiopia, in addition to standard antibiotics and routine care, even with WHO standard oxygen therapy, death from severe pneumonia is very high. Thus, we need to explore improved respiratory/oxygen support and further need to understand whether the improved oxygen support is helpful to reduce these deaths. Oxygen support using bubble continuous positive airway pressure (CPAP) is believed to be one of the improved respiratory supports for children with severe pneumonia who also have lack of oxygen in blood. Recently done research in Bangladesh, Ghana, Malawi, and India for the treatment of children with severe pneumonia with lack of oxygen in blood show that oxygen delivered by bubble CPAP had lower deaths than the WHO standard oxygen treatment. The studies in these countries were done with frequent monitoring. However, we do not know whether this low cost bubble CPAP oxygen therapy will be better as like as in Bangladesh, Ghana, Malawi, and India or worse compared to WHO standard low flow oxygen therapy in urban and rural district hospitals in Ethiopia where we will have less monitoring due to lack of additional manpower.

Thus, our aim is to assess the feasibility and effectiveness of locally constructed oxygen delivery system (bubble CPAP) for the treatment of children with severe pneumonia, which is a low cost oxygen delivery method.

Why you are invited to participate in the study?

Since your child is suffering from severe pneumonia we are inviting you to participate in this study and help us in our efforts to find better treatment of this disease.

Procedures: If you volunteer to allow your child to participate in this study and agree with written informed consent, your child will be given oxygen therapy using bubble CPAP. In addition, your child's socio-demographic and clinical data will be filled on questionnaires by the study nurse. If your child doesn't improve while on bubble CPAP and needs advanced care, he/she will get the care deemed necessary by the treating physician. Your child's involvement will not affect the care your child is entitled to receive and will not have any influence on the decision of the treating physician.

Risks associated with your child's participation in the study: There is no major risk involved in the participation of your child in the study. There will be no difference of standard of care provided to your child in the hospitals. Introducing bubble CPAP is very easy and convenient way to deliver oxygen almost like other currently available (WHO) methods. There is very little chance of any harm from this treatment. Moreover, in the previous efficacy trial of bubble CPAP, there was not a single report of any adverse event from bubble CPAP. It has been practiced widely in the world for many years for treating severe respiratory distress in newborn patients. However, side effects such as nasal trauma, nasal obstruction, gastric distension and pneumothorax are reported to occur in neonates but such complications are found to be extremely rare in older children. In order to minimize those risks all the necessary precautions will be taken and frequent follow up of your child will be done.

Benefits: We do not know whether this low cost bubble CPAP oxygen therapy will be better as in Bangladesh and Ghana or worse compared to WHO standard low flow oxygen therapy in urban and rural district hospitals in Ethiopia. In order to understand the performance of this innovative oxygen therapy in our set up, your child's participation may be helpful for collecting evidence for further implementation of bubble CPAP for treatment of children with severe pneumonia in our setting and at a larger scale.

Outcomes of the study: The findings of this study will provide relevant information on better delivery modality of oxygen for children with severe pneumonia & hypoxia in resource limited settings.

Right to refuse or withdrawal: You have full right to refuse or withdraw your child from participating in this study at any time, and if you wish to do so, this will not affect any of the services your child is entitled to receive. If you refuse on your child's participation your child will be given oxygen via the standard method of oxygen delivery which is low flow oxygen therapy.

Confidentiality: The information of your child related to this study will be kept confidential. Any data related to your child will be kept in a locked cabinet at AHRI and will only be

accessed by the investigators. Any identifier related to your child will be removed if the findings of the study are to be published.
Compensation: You will not be provided with any compensation by letting your child participate in this study.
Contact person: In case of any questions regarding the study or related issues, you can contact any of the following individuals:

Name of the PI: Dr Meseret Gebre Tel: 251-0911-977885, Addis Ababa, Ethiopia
AHRI/ALERT Ethics Review Committee (AAERC), Secretary: Tel: 0118-962183
CHS/AAU Ethics Review Committee, Tel: 0118961396

Annex 2

Participant's parent/caregiver consent form (Stage I & II)

Date: ___________________________

I have been well informed that MOH & AHRI in collaboration with ICDDBR would like to carry out a study on "***Feasibility , acceptability safety, and efficacy/effectiveness of bubble continuous positive airway pressure (CPAP) for treatment of children aged 1-59 months with severe pneumonia in Ethiopia: a cluster randomized controlled clinical trial*** ".

If I agree on my child's participation, I am aware that my child will be given oxygen therapy with bubble CPAP and clinical and demographic data will be collected by the study nurse and that the records will be filled on a questionnaire for the study. I have been also well informed about the voluntary nature of participation and that I can withdraw my child from the study any time. I was informed that my decision won't affect the care my child is entitled to receive. I am also informed that if I refuse to let my child participate in the study my child will get oxygen via the standard of care which is low flow oxygen therapy. In addition, I have been given the information that if my child doesn't improve despite being treated with the locally constructed oxygen delivery system and my child needs advanced care he/she will get all the necessary care based on the decision by the treating physician and my child's involvement in the research won't affect the decision to be made by the treating physician.

The investigator has also explained to me about the risks associated with bubble CPAP and all the necessary precautions will be taken to avoid possible risks.

I got adequate answer to my questions and I have been given enough time to think over allowing the participation of my child in this study before I signed this informed consent. It is therefore with full understanding of the situation that I give my written informed consent for my child to participate in this study.

Name of the parent/care giver: _____________________________Signature ________________

Name of a person taking the consent: _________________________ Signature __________________

Witness: __________________________________Signature ________________

Annex 3. Amharic version of Information sheet (Stage I & II)

በከፍተኛ ሳንባ ምች ለተጠቁ ልጆች ወላጅ /አሳዳጊ የቀረበ የመረጃ ቅ💻

የጥናቱ ር✆ስ፡- በአገር ውስጥ በተሰራ የኦክስጅን መስጫ ዘዴ በመጠቀም በከፍተኛ የሰንባ ምች የተጠቁ ህ☜ናትን ለማከም የቀረበ ጥናት

የጥናቱ አላማ ፡ልጅዎ በሳንባ ምች ህመም የተጠቃ ሲሆን ብዙ ጊዜ የሚከሰተውም በኢንፌክሽን ምክንያት ነው። የሳንባ ምች ከፍተኛ ከሆነ ከሌሎች መድሀኒቶች በተጨማሪ ኦክስጅን መስጠት ሊያስፈልግ ይችላል። ልጅዎ ከሌሎች መድሀኒቶች በተጨማሪ ኦክስጅን ካልተሰጠው በሽታው ተባብሶ ከፍተኛ ደረጃ ሊደርስ ይችላል። በሳንባ ምች ተጠቅቶ/ታ የኦክስጅን እጥረት ላጋጠመው/ማት ህ☜ን የተለያዩ የኦክስጅን መስጫ መንገዶች ሲኖሩ የአለም ጤና ድርጅት የአፍንጫ ቱቦ ከኦክስጅን ሲሊንደር ጋር በማገናኘት እንዲሰጥ ይመክራል። ነገር ግን በባንግላዲሽ የተደረገ ጥናት እንዳሳየው በሳንባ ምች ለተጠቁና የኦክስጅን እጥረት ላገጠማቸው ህ☜ናት ኦክስጅን በበብል ሲፓፕ (ጠቀሜታው የተሻለ ተብሎ ነው የሚታሰብ በቀላሉ የሚገጣጠም መሳሪያ) ቢሰጥ የተሻለ መሆኑን አሳይቶአል።ጥናቱም የተካሄደው በከፍተኛ ክትትልና ተጨማሪ የሰው ሃይል በማካተት ነው። ይህንንም መሳሪያ በሀገራችን ለሚገኙ በሳንባ ምች ለተጠቁ ህጻናት ለመጠቀም እንዲቻል ጥናቱን አነስተኛ የሰው ሀይል ባለባቸው ሆስፒታሎች መስራት አስፈላጊ ነው።ምክንያቱም ይህንን መሳሪÁ አነስተኛ የሰው ሀይል እና ክትትል ባለባቸው ሆስፒታሎች ¨<eØ K=c^ እ”ÅT>‹M T[ÒÑØ ÁeðMÒM:: በመሆኑም በዚህ ጥናት ይህንን መሳሪያ በኢትዮጵያ ለሚገኙ በከፍተኛ የሳንባ ምች ለተጠቁ ህ☜ናት ለማድረስ ሊያጋጥሙ የሚችሉ ተግዳሮቶችን ጥናት እናደርጋለን።

በጥናቱ እንዲሳተፉ የተጠየቁበት ምክንያት

ልጅዎ በከፍተኛ በሳንባ ምች ስለተጠቃና ለዚህም ህመም የተሻለ ህክምና ለማጥናት ይረዱን ዘንድ ነው።

የጥናቱ ሂደት፡- በጥናቱ ውስጥ ልጅዎ እንዲሳተፍ ከፈቀዱና በስምምነት ቅ💻 ላይ ከፈረሙ ልጅዎ አገር ውስጥ በተገጣጠመው የኦክስጅን መስጫ በብል ሲፓፕ (ጠቀሜታው የተሻለ ተብሎ ነው የሚታሰብ በቀላሉ የሚገጣጠም መሳሪያ) ኦክስጅን ይሰጠዋል በተጨማሪም የልጅዎ ማህበራዊ እና ሌሎች የህክምና መረጃወች ቅ💻 ላይ በጥናቱ ነርስ አማካኝነት ይሞላል። ልጅዎ በዚህ መሳሪያ እየታከመ የማይaለው እና ከፍተኛ ህክምና የሚያስፈልገው ከሆነ በሀኪሙ አማካኝነት አስፈላጊው ህክምና ይደረግለታል። የልጅዎ በጥናቱ ላይ መሳተፍ ሃኪሙ በሚወስነው ውሳኔ ላይ ምንም አይነት ተ💻ዕኖ አይኖረውም።

ስጋትና ጉዳት፡- ይህ የኦክስጅን አሰጣጥ ቀላል የሚባል ሲሆን ከባድ የሚባል ጉዳት ያደርሳል ተብሎ አይጠበቅም።በተጨማሪም በፊት በባንግላዲሽ ጋና እንዲሁም ኡጋንዳ በተደረጉ ጥናቶች ላይ ምንም አይነት ጉዳት አልተከሰተም።ነገር ግን አልፎ አልፎ

የአፍንጫ መቁሰል🔔የአፍንጫ መደፈን የሆድ መወጠርና በሳንባ ሽፋን ውስጥ አየር መሞላት የመሳሰሉት ጉዳት በጨቅላ ህ☜ናት ላይ ሊከሰቱ እንደሚችሉ የተጠቀሰ ቢሆንም እድሜአቸው ከፍ ባሉ ህ☜ናት ላይ የመከሰት እድሉ ግን እጅግ በጣም አናሳ ነው

እነዚህንም ጉዳቶች ለመከላከል አስፈላጊው ጥንቃቄ እና ክትትል ይደረጋል

በጥናቱ መሳተፍ ያለው ጠቀሜታ፡-ልጅዎ በዚህ ጥናት መሳተፉ

የዚህ የኦክስጅን አሰጣጥ ዘዴ ጠቀሜታ በጥናት ለማሳየት ተጨማሪ መረጃ ለመሰብሰብና በተመሳሳይ ችግር ለሚጠቁ ህ☜ናት በሰፊው ለመተግበር የሚያስችል መረጃ ያስገኛል።

የጥናቱ ውጤት፡-ዝቅተኛ ገቢ ባላቸው ሀገሮች ውስጥ የዚህ ጥናት ውጤት በከፍተኛ የሳንባ ምች ለታመሙ ህ☜ናት የተሻለ የኦክስጅን አሰጣጥን በተመለከተ ጠቃሚ መረጃ ይሰጣል።

በጥናቱ አለመሳተፍ/ፍቃደኛ ያለመሆን፡- በዚህ ጥናት ውስጥ ልጅዎ እንዳይሳተፍ የማድረግ ወይም ከጀመሩ በኋላ የማቋረጥ ሙሉ መብት አልዎት ይህንን ማድረግዎ ልጅዎ ማግኘት የሚገባው/ት ህክምና ላይ ምንም አይነት ተ💻✆ኖ አያመጣም። ልጅዎ በጥናቱ ላይ እንዳይሳተፍ ከወሰኑ ልጅዎ በተለመደው የኦክስጅን መስጫ መሳሪያ ኦክስጅን ይሠጠዋል።

የጥናቱ ሚስጥራዊነት፡-ልጅዎን የተመለከተ ማንኛውም መረጃ ሚስጥራዊነቱ የተጠበቀ ነው

የልጅዎ መረጃ በአህሪ በተቆለፈ ሳጥን ውስጥ ይቀመጣል መረጃውን ሊያገኙ የሚችሉት አጥኚዎቹ ብቻ ናቸው ልጅዎን የተመለከተ መረጃ ለህትመት የሚበቃ ከሆነ የልጅዎን ማንነት የሚገልጡ መረጃዎች በሙሉ ይሰረዛሉ

በዚህ ጥናት ልጅዎ በመሳተፉ ምንም አይነት ክፍያ አይኖርም

ስለጥናቱ ጥያቄ ካለዎት የጥናቱን ዋና ተመራማሪ ዶ/ር መሰረት ገብሬ ስ.ቁ 0911977885 ወይም

የአህሪ ኢቲክስ ኮሚቴ ስ.ቁ 0118962183 ማነጋገር ይችላሉ

የአዲስ አበባ ዩኒቨርሲቲ ኢቲክስ ኮሚቴስ.ቁ 0118961396

Annex 4. Amharic version of Consent form (Stage I & II)

የተሳታፊ ህጻናት ወላጅ/ አሳዳጊ የስምምነት ቅ💻

ቀን---------------------------

የጤና ጥበቃ ድርጅት   እና   አህሪ hiddr, b ጋር በመተባበር በብል ሲፓፕ የተባለውን ኦክስጂን መስጫ በመጠቀም የሳንባ ምች ያለባቸውን ህ ☜ናት ማከም

በሚል ርዕስ ላይ ጥናት ለመስራት እንዳቀዱ ተገልጦልኛል

ልጄ በጥናቱ ላይ እንዲሳተፍ ከተስማማሁ ለልጄ ኦክስጅን የሚሰጠው በብል ሲፓፕ በተባለው መሳሪያ እንደሆነ ተገልጦልኛል እንዲሁም ልጄን የተመለከቱ ማህበራዊና የጤንነት መረጃዎች በጥናቱ ነርስ አማካይነት እንደሚወሰድ ተነግሮኛል ልጄ በዚህ መሳሪያ እየታከመ የማይaለው እና ከፍተኛ ህክምና የሚያስፈልገው ከሆነ በሀኪሙ አማካኝነት አስፈላጊው ህክምና እንደሚደረግለት እንዲሁም የልጄ በጥናቱ ላይ መሳተፍ ሃኪሙ በሚወስነው ውሳኔ ላይ ምንም አይነት ተ💻ዕኖ እንደማይኖረው ተገልፀልኛል።

በተጨማሪም በጥናቱ ላይ መሳተፍ በፈቃደ ነት ላይ የተመሰረተ እንደሆነ ተነግሮኛል ልጄንም በማንገኛውም ጊዜ ከጥናቱ

እንዲያ ቋርጥ ማድረግ እንደምችል ተነግሮኛል የምወስነውም ውሳኔ ልጄ በሚያገኘው ህክምና ላይ ምንም ተ💻እኖ እንደማያመጣ ተረድቻለሁ ልጄ በጥናቱ ላይ ከወሰንኩ በተለመደው የኦክስጅን መስጫ መሳሪያ ኦክስጅን እንደሚሰጠው ተገልፀልኛል።

እንዲሁም በብል ሲፓፕ ሊያመጣው የሚችለውን የጎን ጉዳትና እንዳይከሰትም አስፈላጊው ጥንቃቄ እንደሚደረግ ተገልፀልኛል

ለጠየቅኩት ጥያቄዎች በቂ ምላb ካገኘሁ እና በጉዳዩ ላይ ለማሰብ በቂ ጊዜ ከተሰጠኝ በኋላ በሙሉ መረዳት ልጄ በጥናቱ ላይ እንዲሳተፍ ፈቃደኝነቴን በፊርማዬ አረጋግጫለሁ

የአሳዳጊ/ወላጅ ስም-------------   ፊርማ

ፈቃደኝነት የሚያስፈርመው ስም--------------   ፊርማ

የምስክር ስም--------------   ፊርማ

Annex5. Information sheet for Parents/Guardians of children with Severe pneumonia (Stage III)

Title of the study*: Feasibility, acceptability, safety, and efficacy/effectiveness of bubble continuous positive airway pressure (CPAP) for treatment of children Aged 1-59 months with severe pneumonia in Ethiopia: a cluster randomized controlled clinical trial*

Purpose of the research

Pneumonia is a disease caused by infection in lungs. Pneumonia may become severe and require oxygen therapy along with other medication. There are different forms of delivering oxygen to a child with severe pneumonia. Our aim is to assess the feasibility and effectiveness of locally constructed oxygen delivery system (bubble CPAP) for the treatment of children with severe pneumonia, which is a low cost oxygen delivery method.

Procedures: If you volunteer to allow your child to participate in this study and agree with written informed consent, your child will be randomly assigned to oxygen therapy using bubble CPAP or low flow oxygen therapy which is the standard of care. In addition, your child's socio-demographic and clinical data will be filled on questionnaires by the study nurse. If your child doesn't improve while on bubble CPAP or low flow oxygen therapy and needs advanced care, he/she will get the care deemed necessary by the treating physician. Your child's involvement will not affect the care your child is entitled to receive and will not have any influence on the decision of the treating physician.
In case you decide to leave with your child against medical advice during any time of your stay, we will contact you via phone or physically to know the final outcome of your child.

Risks to study participants:

BCPAP

Although side effects such as nasal trauma, partial nasal obstruction, gastric distension and pneumothorax are reported to occur while using bubble CPAP in neonates, and such complications are found to be extremely rare in older children from studies in Bangladesh, Ghana, Malawi, and India, we do not know its incidence in children with severe pneumonia beyond newborn period who will receive bubble CPAP in Ethiopia. If there is any side effects in Ethiopia, in order to minimize those risks all the necessary precautions will be taken and frequent follow up with routine monitoring of vital signs of your child will be done.

Low flow (LF)

This oxygen delivery mechanism is simple and is not expected to result in major complication. But occasionally nasal trauma, partial nasal obstruction and abdominal distension may occur. But all the necessary precautions and follow up will be done to prevent the problems.

Benefits to study participants: Your child's participation is highly beneficial for collecting evidence for further implementation of bubble CPAP for treatment of children with severe pneumonia at a larger scale.

Outcomes of the study: The findings of this study will provide relevant information on better delivery modality of oxygen for children with severe pneumonia & hypoxaemia in resource limited settings.

Right to refuse or withdrawal: You have full right to refuse or withdraw your child from participating in this study at any time, and if you wish to do so, this will not affect any of the services your child is entitled to receive.

Confidentiality: The information of your child related to this study will be kept confidential. Any data related to your child will be kept in a locked cabinet at AHRI and will only be accessed by the investigators. Any identifier related to your child will be removed if the findings of the study are to be published.

Compensation: You will not be provided with any compensation by letting your child participate in this study.

Contact person: In case of any questions regarding the study or related issues, you can contact any of the following individuals:

Name of the PI: Dr Meseret Gebre Tel: 251-0911-977885, Addis Ababa, Ethiopia
AHRI/ALERT Ethics Review Committee (AAERC), Secretary: Tel: 251-0118-962183

Annex 6. Participant's parent/caregiver consent form (Stage III)

Date: ___________________________

I have been well informed that MOH & AHRI in collaboration with icddrb would like to carry out a study on "***Feasibility , acceptability, safety, and efficacy/effectiveness of bubble continuous positive airway pressure (CPAP) for treatment of children Aged 1-59 months with severe pneumonia in Ethiopia: a randomized controlled clinical trial*** ".

If I agree on my child's participation, I am aware that my child will be randomly assigned to oxygen therapy via bubble CPAP or standard low flow oxygen therapy and clinical and demographic data will be collected by the study nurse and that the records will be filled on a questionnaire for the study. I have been also well informed the voluntary nature of participation and that I can withdraw my child withdraw from the study any time. I was informed that my decision won't affect the care my child is entitled to receive.

I am also informed that if I refuse to let my child participate in the study my child will get oxygen via the standard of care which is low flow oxygen therapy. In addition, I have been given the information that if my child doesn't improve despite being treated with the locally constructed oxygen delivery system or low flow oxygen therapy and my child needs advanced care he/she will get all the necessary care based on the decision by the treating physician and my child's involvement in the research won't affect the decision to be made by the treating physician.

The investigator has also explained to me about the risks associated with bubble CPAP and all the necessary precautions will be taken to avoid possible risks. I have also been informed that In case I decide to leave with my child against medical advice during any time of my stay, I will contacted via phone or physically to know the final outcome of my child.

I have been given enough time to think over allowing the participation of my child in this study before I signed this informed consent. It is therefore with full understanding of the situation that I give my written informed consent for my child to participate in this study.

Name of the parent/care giver: _____________________________Signature _______________

Name of a person taking the consent: _________________________ Signature _________________

Witness: __________________________________Signature ________________

Annex 7. Amharic version of Information sheet (Stage III)

በከፍተኛ ሳንባ ምች ለተጠቁ ልጆች ወላጅ /አሳዳጊ የቀረበ የመረጃ ቅ💻

<u>የጥናቱ አላማ </u>፡ልጅዎ በሳንባ ምች ህመም የተጠቃ ሲሆን ብዙ ጊዜ የሚከሰተውም በኢንፌክሽን ምክንያት ነው። የሳንባ ምች ከፍተኛ ከሆነ ከሌሎች መድሀኒቶች በተጨማሪ ኦክስጅን መስጠት ሊያስፈልግ ይችላል። ልጅዎ ከሌሎች መድሀኒቶች በተጨማሪ ኦክስጅን ካልተሰጠው በሽታው ተባብሶ ከፍተኛ ደረጃ ሊደርስ ይችላል። በሳንባ ምች ተጠቅቶ/ታ የኦክስጅን እጥረት ላጋጠመው/ማት ህ☜ን የተለያዩ የኦክስጅን መስጫ መንገዶች ሲኖሩ የአለም ጤና ድርጅት የአፍንጫ ቱቦ ከኦክስጅን ቐት ጋር በማገናኘት እንዲሰጥ ይመክራል። ነገር ግን በባንግላዲሽ የተደረገ ጥናት እንዳሳየው በሳንባ ምች ለተጠቁና የኦክስጅን እጥረት ለገጠማቸው ህ☜ናት ኦክስጅን በበብል ሲፓፕ(ጠቀሜታው የተሻለ ተብሎ ነው የሚታሰብ በቀላሉ የሚገጣጠም መሳሪያ) ቢሰጥ የተሻለ መሆኑን አሳይቶአል።ጥናቱም የተካሄደው በከፍተኛ ክትትልና ተጨማሪ የሰው ሃይል በማካተት ነው። ይህንንም መሳሪያ በሀገራችን ለሚገኙ በሳንባ ምች ለተጠቁ ህጻናት ለመጠቀም እንዲቻል ጥናቱን አነስተኛ የሰው ሀይል ባለባቸው ሆስፒታሎች መስራት አስፈላጊ ነው።ምክንያቱም ይህንን መሳሪያ አነስተኛ የሰው ሀይል እና ክትትል ባለባቸው ሆስፒታሎች ውስጥ ሊሰራ እንደሚችል ማረጋገጥ ያስፈልጋል:::: በመሆኑም በዚህ ጥናት ይህንን መሳሪያ በኢትዮጵያ ለሚገኙ በከፍተኛ የሳንባ ምች ለተጠቁ ህ☜ናት ለማድረስ ሊያጋጥሙ የሚችሉ ተግዳሮቶችን ጥናት እናደርጋለን።

በጥናቱ እንዲሳተፉ የተጠየቁበት ምክንያት

ልጅዎ በከፍተኛ በሳንባ ምች ስለተጠቃና ለዚህም ህመም የተሻለ ህክምና ለማጥናት ይረዱን ዘንድ ነው።

<u>የጥናቱ ሂደት፡-</u> በጥናቱ ውስጥ ልጅዎ እንዲሳተፍ ከፈቀዱና በስምምነት ቅ💻 ላይ ከፈረሙ ልጅዎ አገር ውስጥ በተገጣጠመው የኦክስጅን መስጫ በብል ሲፓፕሲፓፕ(ጠቀሜታው የተሸለ ለው ተብሎ የሚታሰብ በቀላሉ ጠሚገጣም መሳሪያ) ወይም በተለመደው የኦክስጅን አሰጣጥ ኦክስጅን ይሰጠዋል በተጨማሪም የልጅዎ ማህበራዊ እና ሌሎች የህክምና መረጃዎች ቅ💻 ላይ በጥናቱ ነርስ አማካኝነት ይሞላል።ልጅዎ በዚህ መሳሪያ ወይም በተለመደው የኦክስጅን መስጫ እየታከመ የማይሻለው እና ከፍተኛ ህክምና የሚያስፈልገው ከሆነ በሀኪሙ አማካኝነት አስፈላጊው ህክምና ይደረግለታል። የልጅዎ በጥናቱ ላይ መሳተፍ ሃኪሙ በሚወስነው ውሳኔ ላይ ምንም አይነት ተ💻ዕኖ አይኖረውም። ምናልባት ልጅዎን ከሃኪም ፈቃድ ውጪ ይዘው ለመሄድ ቢወስኑ የልጅዎን የመጨረሻ ውጤት ለማወቅ አንድ የጥናቱ ቡድን አባል በስልክ አሊያም በአካል ያገኝዎታል።

ስጋትና ጉዳት<u>፡-</u>

በብል ሲፓፕ፡

ይህ የኦክስጅን አሰጣጥ ቀላል የሚባል ሲሆን ከባድ የሚባል ጉዳት ያደርሳል ተብሎ አይጠበቅም።በተጨማሪም በፊት በባንግላዲሽ ጋና እንዲሁም ኡጋንዳ በተደረጉ ጥናቶች ላይ ምንም አይነት ጉዳት አልተከሰተም።ነገር ግን አልፎ አልፎ

የአፍንጫ መቁሰል፣የአፍንጫ መደፈን የሆድ መወጠርና በሳንባ ሽፋን ውስጥ አየር መሞላት የመሳሰሉት ጉዳት በጨቅላ ህፃናት ላይ ሊከሰቱ እንደሚችሉ የተጠቀሰ ቢሆንም እድሜአቸው ከፍ ባሉ ህፃናት ላይ የመከሰት እድሉ ግን እጅግ በጣም አናሳ ነው

እነዚህንም ጉዳቶች ለመከላከል አስፈላጊው ጥንቃቄ እና ክትትል ይደረጋል

የተለመደው የኦክስጂን አሰጣጥ(low flow)

ይህ የኦክስጂን አሰጣጥ ቀላል የሚባል ሲሆን ከባድ የሚባል ጉዳት ያደርሳል ተብሎ አይጠበቅም።ነገር ግን አልፎ አልፎ የአፍንጫ መቁሰል ፣የአፍንጫ በከፊል መደፈን እንዲሁም የሆድ መወጠር ሊከሰት ይችላል፡፤ነገር ግን እነዚህን ጉዳቶች ለመከላከል አስፈላጊው ጥንቃቄ እና ክትትል ይደረጋል

በጥናቱ መሳተፍ ያለው ጠቀሜታ፡-ልጅዎ በዚህ ጥናት መሳተፉ

የዚህ የኦክስጅን አሰጣጥ ዘዴ ጠቀሜታ በጥናት ለማሳየት ተጨማሪ መረጃ ለመሰብሰብና በተመሳሳይ ችግር ለሚጠቁ ህፃናት በሰፊው ለመተግበር የሚያስችል መረጃ ያስገኛል።

የጥናቱ ውጤት፡-ዝቅተኛ ገቢ ባላቸው ሀገሮች ውስጥ የዚህ ጥናት ውጤት በከፍተኛ የሳንባ ምች ለታመሙ ህፃናት የተሻለ የኦክስጅን አሰጣጥን በተመለከተ ጠቃሚ መረጃ ይሰጣል።

በጥናቱ አለመሳተፍ/ፍቃደኛ ያለመሆን፡- በዚህ ጥናት ውስጥ ልጅዎ እንዳይሳተፍ የማድረግ ወይም ከጀመሩ በኋላ የማቋረጥ ሙሉ መብት አልዎት ይህንን ማድረግዎ ልጅዎ ማግኘት የሚገባው/ት ህክምና ላይ ምንም አይነት ተ[illegible]ኖ አያመጣም።

የጥናቱ ሚስጥራዊነት፡-ልጅዎን የተመለከተ ማንኛውም መረጃ ሚስጥራዊነቱ የተጠበቀ ነው

የልጅዎ መረጃ በአህሪ በተቆለፈ ሳጥን ውስጥ ይቀመጣል መረጃውን ሊያገኙ የሚችሉት አጥኚዎቹ ብቻ ናቸው ልጅዎን የተመለከተ መረጃ ለህትመት የሚበቃ ከሆነ የልጅዎን ማንነት የሚገልጡ መረጃዎች በሙሉ ይሰረዛሉ

በዚህ ጥናት ልጅዎ በመሳተፉ ምንም አይነት ክፍያ አይኖርም

ስለጥናቱ ጥያቄ ካለዎት የጥናቱን ዋና ተመራማሪ ዶ/ር መሰረት ገብሬ ስ.ቁ 0911977885 ወይም

የአህሪ ኢቲክስ ኮሚቴ ስ.ቁ 0118962183 ማነጋገር ይችላሉ

Annex 8. Amharic version of consent form (Stage III)

የተሳታፊ ህጻናት ወላጅ/ አሳዳጊ የስምምነት ቅ💻

ቀን---------------------------

የጤና ጥበቃ ድርጅት እና አህሪ ከICDDRB ጋር በመተባበር በብል ሲፓፕ የተባለውን ኦክስጂን መስጫ በመጠቀም የሳንባ ምች ያለባቸውን ህ ☜ናት ማከም

በሚል ርዕስ ላይ ጥናት ለመስራት እንዳቀዱ ተገልጦልኛል

ልጄ በጥናቱ ላይ እንዲሳተፍ ከተስማማሁ ለልጄ ኦክስጅን የሚሰጠው በብል ሲፓፕ በተባለው መሳሪያ ወይም በተለመደው የኦክስጅን አሰጣጥ እንደሆነ ተገልጦልኛል እንዲሁም ልጄን የተመለከቱ ማህበራዊና የጤንነት መረጃዎች በጥናቱ ነርስ አማካይነት እንደሚወሰድ ተነግሮኛል ልጄ በዚህ መሳሪያ ወይም በተለመደው የኦክስጅን መስጫ እየታከመ የማይሻለው እና ከፍተኛ ህክምና የሚያስፈልገው ከሆነ በሀኪሙ አማካኝነት አስፈላጊው ህክምና እንደሚደረግለት እንዲሁም የልጄ በጥናቱ ላይ መሳተፍ ሃኪሙ በሚወስነው ውሳኔ ላይ ምንም አይነት ተ💻ዕኖ እንደማይኖረው ተገልጾልኛል።

በተጨማሪም በጥናቱ ላይ መሳተፍ በፈቃደ ነት ላይ የተመሰረተ እንደሆነ ተነግሮኛል ልጄንም በማንገኛውም ጊዜ ከጥናቱ እንዲያ ቋርጥ ማድረግ እንደምችል ተነግሮኛል የምወስነውም ውሳኔ ልጄ በሚያገኘው ህክምና ላይ ምንም ተ💻እኖ እንደማያመጣ ተረድቻለሁ

እንዲሁም በብል ሲፓፕ ወይም የተለመደው የኦክስጅን አሰጣጥ ሊያመጣው የሚችለውን የጎን ጉዳትና እንዳይከሰቱም አስፈላጊው ጥንቃቄ እንደሚደረግ ተገልጦልኛል::ምናልባት ልጄን ከሃኪም ፈቃድ ውጪ ይዠ ለመሄድ ብወስን የልጄን የመጨረሻ ውጤት ለማወቅ አንድ የጥናቱ ቡድን አባል በስልክ አሊያም በአካል እንደሚያገኘኝ ተነግሮኛል።

በጉዳዩ ላይ ለማሰብ በቂ ጊዜ ከተሰጠኝ በኋላ በሙሉ መረዳት ልጄ በጥናቱ ላይ እንዲሳተፍ ፈቃደኝነቴን በፊርማዬ አረጋግጫለሁ

የአሳዳጊ/ወላጅ ስም-------------   ፊርማ -----------------   ቀን --------
----

አሳዳጊው ከጥናቱ ተሳታፊ ጋር ያለው ግንኙነት ----------------

ፈቃደኝነት የሚያስፈርመው ስም--------------   ፊርማ ----------------ቀን---------
----

የምስክር ስም--------------   ፊርማ------------------------ቀን------

Annex 9: Questionnaire

Date: DD/MM/YY

Subject Identification, Clinical and Physical Examination Form

Title: ***Feasibility, acceptability, safety, and efficacy/effectiveness of bubble continuous positive airway pressure (CPAP) for treatment of children Aged 1-59 months with severe pneumonia in Ethiopia: a cluster randomized controlled clinical trial***

Study code: ______________________ Name: _________________

Socio-demographic characteristics:

1. Maternal education

a. illiterate

b. read & write

c. <6 grade

d. 7-8 grade

e. 9-12 grade

f. graduate

2. Family size

a. <5

b. 5-10

c.>10

3. Monthly income

a. <500br

b. 500-1000 br

c. 1000-2000br

d. >2000br

e. house hold assets

1. Sex: Male< ☐ Female ☐ Age (yrs):__________ Weight (kg): ___________ Height (cm): _
2. History of presenting illness: Cough -----
   Fever ------
   Fast breathing/SOB-----
   Others specify------
3. Duration of illness in days------
4. New------------Repeated---------- date of last admission-------
5. Clinical findings

**Parameter**	**Admission**	**1 hour**	**6 hours**	**12 hours**	**24 hours**	**48 hours**	**72 hours**
Respiratory rate							
Nasal flaring							
Chest indrawing							
Grunting							
Cyanosis							
Consciousness(GCS)							
Oxygen saturation							
Temperature							

Heart rate							

5. CXR finding---

6. HIV status
   a. Negative
   b. Positive
   c. Exposed
   d. Unknown

8. Type of antibiotic started ------

9. Length of hospital stays in days **-------**

10. Length of stay on bubble CPAP in days--------

11. Outcome of the patient?
   a. Intubated and put on mechanical ventilation
   b. Improved and discharged
   c. Died
   d. Discharged against medical advice

12. Any complications noted

a. Nasal trauma yes No

b. Nasal obstruction yes No

c. Gastric distension yes No

d. Pneumothorax yes No

Annex 10. Constructing local bCPAP

The bubble CPAP, made in Bangladesh, will be reproduced in Ethiopia during the study period. The height of the bubble CPAP bottle (filled with water during its use for the patient) is 17 cm, radius is 17 cm and diameter is 7.5 cm. Length of the tubing from the oxygen source up to the water filled plastic bottle ranges from 230 to 300 cm.

Appropriate sized nasal prong (such as infant size for 1-12 months and pediatric size for >12 months) will be used for each child which will fit to the child's nostrils. One of the limbs of the nasal prong will be cut and the distal end will be tied up. The proximal limb will be connected to IV fluid tubing which will then be inserted to a graduated water bottle. The depth of the inserted tube into the water will gauge the amount of pressure to be delivered. The depth will start from 5 cm. which can be increased up to 8 cm. depending on the child's response.

The main distal limb of the nasal prong will be connected to oxygen source which could be either an oxygen cylinder or a concentrator. An oxygen concentrator (shown in the figure below) will be used as one of the sources of oxygen to run this locally made low cost innovative bubble CPAP. Oxygen concentrator is assembled in the USA and the concentrator is compatible with cylinder trans-fill systems. The 1025 unit, with a weight of 42 lbs. is capable of delivering 87% to 96% pure O_2 from 2 to 10 liters per minute, and the concentrator can relentlessly deliver oxygen for 2-3 days and after few hours pause it can again deliver oxygen for same duration with same flow.

The possible side effects of the device from previous studies include nasal trauma, nasal congestion, abdominal distension and rarely pneumothorax but the incidence of these events were found to be same as with WHO standard low flow oxygen therapy [10].

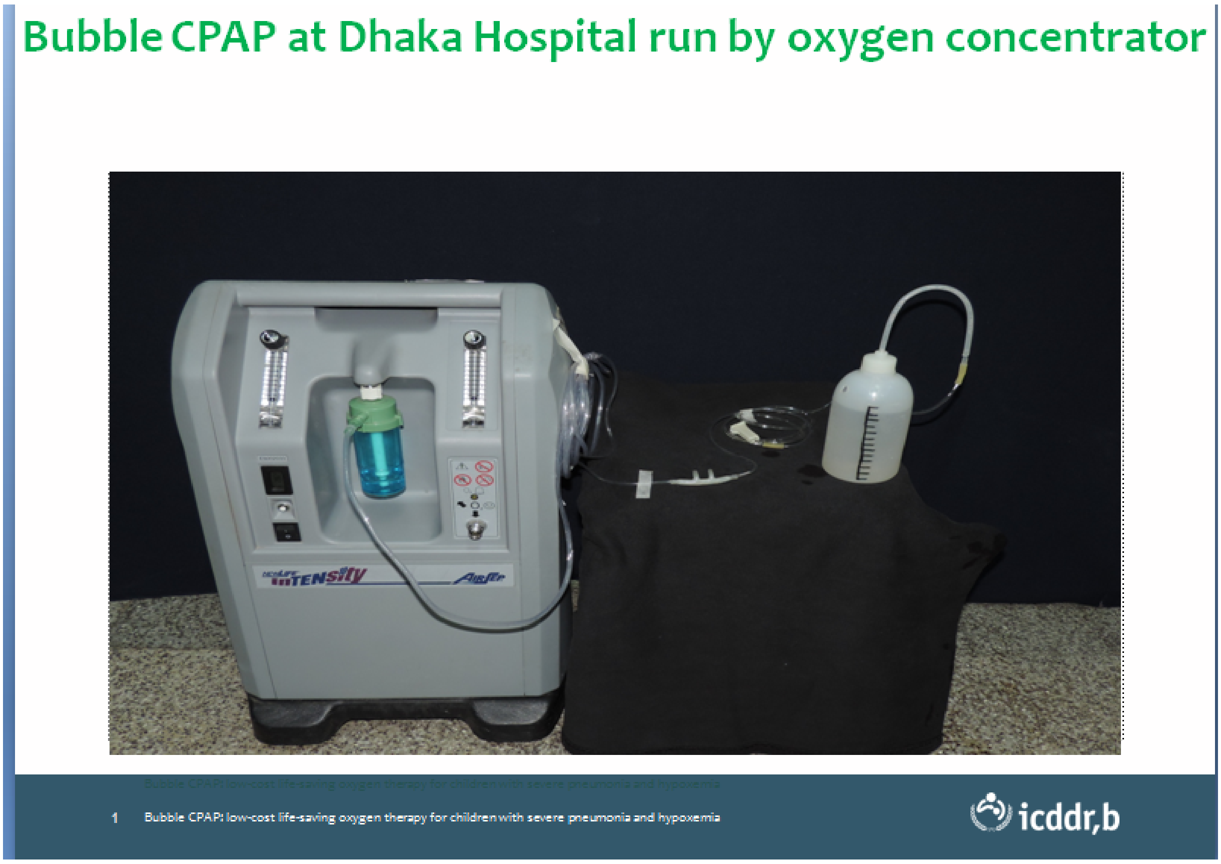
Bubble CPAP at Dhaka Hospital run by oxygen concentrator
INTENSITY
AirSep
1
Bubble CPAP: low-cost life-saving oxygen therapy for children with severe pneumonia and hypoxemia
icddr,b

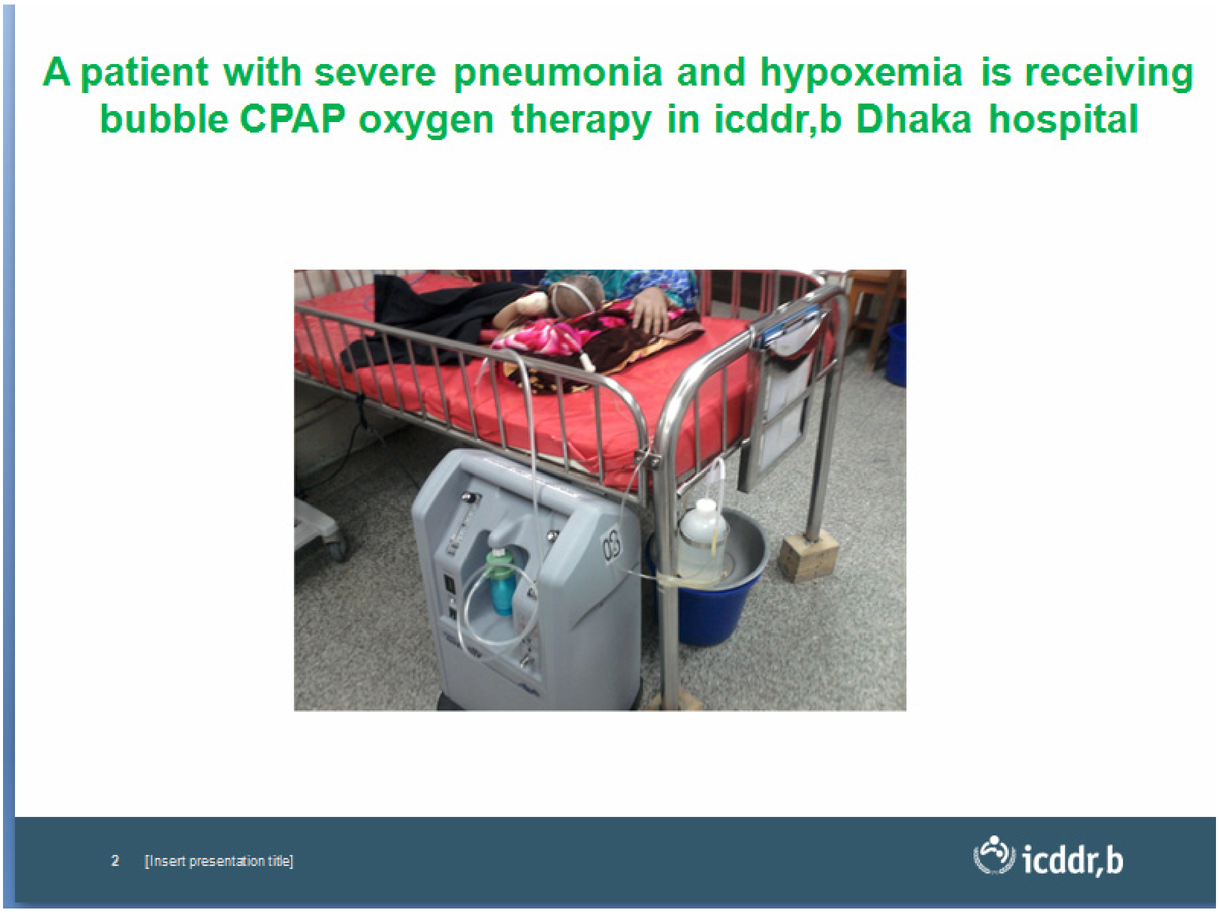

Annex 11. Questions for focus group discussion & In-depth interview guide.

- How easy is constructing local bubble CPAP?
- Acceptability of locally constructed bubble CPAP by health workers
- Cost implication of bubble CPAP
- Sustaining bubble CPAP
- Challenges of scaling up bubble CPAP nationally

Annex 12. Role of Investigators

Dr. Mohammod Jobayer Chisti, MBBS, MMed, PhD, Senior Scientist & Head, Clinical Research, Hospitals, Nutrition and Clinical Services Division (NCSD) and Clinical Lead, ICU, Dhaka Hospital at icddr,b

-will be one of the PIs (Bangladesh) of this project and implement the project in Ethiopian hospitals. He will also be involved with development and various approvals of the project. He will be responsible for overall supervision of the project, co-ordination with the investigators and training of study personnel in collaborative sites. He will contribute to data management, analyses, and report writing and dissemination activities.

Dr. Meseret Gebre, MD, pediatrician, ALERT centre

-will be one of the PIs (Ethiopia) of this project and implementing the project in Ethiopian hospitals. She will also be involved in the development and approval of the project. She will be responsible for overall supervision of the project, co-ordination with the investigators and training of study personnel in collaborative sites. She will contribute to data management, analyses, and report writing and dissemination activities.

Professor John D Clemens, MD, Executive Director, icddr,b

-will be the Co-PI of this project. He will also be involved with development and approval of the project. He will also be responsible for supervision of the project and co-ordination with the sites. Additionally, he will also contribute to data management, analyses, and report writing and dissemination activities.

Professor Tahmeed Ahmed, MBBS, PhD, Senior Director, NCSD, icddr,b

-will be the Co-PI of this project. He will also be involved with development and approval of the project. He will also be responsible for supervision of the project and co-ordination with the sites. Additionally, he will also contribute to data management, analyses, and report writing and dissemination activities.

Professor Trevor Duke, MD, FRACP, Director, Centre for International Child Health

- will be the Co-PI of this project. He will also be involved with development and approval of the project. He will also be responsible for supervision of the project. Additionally, he will also contribute to data management, analyses, and report writing and dissemination activities.

Dr Abebe Genetu Bayih (PhD) ***Director General, AHRI***

-Is responsible for the overall leadership of the research project including securing funds and coordination the Bangladesh and Ethiopian team.

Meles Solomon (MSC) ***Child Health Expert, Maternal and child health directorate, Federal Ministry of health***

-Will be responsible for communicating with the regional health bureaus and selection of research team at the primary hospitals.

-Will be responsible for supervision of research sites at primary hospitals after start of the research project.

Dr Abate Yeshidinber ***Assistant Professor of Pediatrics and Child Health, St. Paulos Milleneum Medical College***

-Will be responsible for coordinating proper conduct of the research project at Saint Pauls' Milleneum Medical College.

--Will be responsible for supervision of research sites at primary hospitals after start of the research project.

Dr Rahel Argaw (MD), ***Assistant Professor of Pediatrics and Child Health, Pulmonology & Critical Care Fellow, TASH***

-Will be responsible for coordinating proper conduct of the research project at Tikur Anbessa Specialized Hospital.

-Will be responsible for supervision of research sites at primary hospitals after start of the research project.

Professor Bogale Worku ***Professor of Pediatrics and Child Health, Executive Director of the Ethiopian Pediatrics Society***

-Will lead the technical preparation of the locally constructed bubble CPAP.

-Will be responsible for facilitating training of data collectors & site supervisors.

-Will be responsible for supervision of research sites at primary hospitals after start of the research project.

Dr Asrat Demtse (MD), ***Assistant Professor of Pediatrics and Child Health, Neonatologist***

-Will lead the technical preparation of the locally constructed bubble CPAP.

-Will be responsible for facilitating training of data collectors & site supervisors.

-Will be responsible for supervision of research sites at primary hospitals after start of the research project.

Annex 13. List of selected hospitals

St. Paul’s Hospital
Black Lion Hospital<br><br>Yekatit 12 Hospital
Tirunesh Beijing General Hospital
Tulubolo General Hospital
Dil Chora General Hospital
Woliso General Hospital (Missionary Hospital)
Fiche General Hospital
Butajira General Hospital
Worabe Specialized Hospital
Sabian General Hospital
Durame General Hospital
Ziway Memorial Hospital, Addis
Shegaw Motta General Hospital, Addis
Hawassa Adare General Hospital

Annex 14

Management algorithm for children 1-59 months with acute respiratory infections

Step-1 History and examinations:

Clinical history	Examination
• H/O cough or breathing difficulties<br>• H/O inability to drink/ breast fed<br>• H/O convulsion	• Appearance/ mentation<br>• Respiratory rate<br>• Heart rate<br>• Temperature<br>• Dehydration status<br>• Respiratory effort (e.g chest wall indrawing, granting respiration<br>• Cyanosis<br>• SPO2<br>• Chest auscultation (breath sound/ added sound)

On the basis of the above history and examination diagnosis of pneumonia as per following criteria:

Step-2 Diagnosis of pneumonia:

Signs and symptoms	Diagnosis
• Cough or difficulty in breathing with:<br>- Oxygen saturation < 90% or central cyanosis, OR,<br>- grunting), OR<br>- any of the danger signs:<br>➢ Inability to breastfeed or drink<br>➢ Lethargy or reduced level of consciousness<br>➢ Convulsions	Severe pneumonia
• Fast breathing:<br>– ≥ 50 breaths/min in a child aged 2–11 months<br>– ≥ 40 breaths/min in a child aged 1–5 years<br>• Chest indrawing	Pneumonia
■ No signs of pneumonia or severe pneumonia	No pneumonia: cough or cold

Pneumonia

– Home care
– Give appropriate Antibiotic (oral amoxicillin)
– Advise the mother/caregiver when to return immediately if symptoms of severe pneumonia.
– Follow up after 3 days.

No pneumonia

– Home care
– Soothe the throat and relieve cough with safe remedy (e.g honey with warm water)
– Advise the mother when to return.
– Follow up after 5 days if not improving
– If coughing for more than 14 days, evaluation for the etiology of chronic cough

Follow up of the patients at hospital who are on treatment for severe pneumonia

Step-4 Follow up:

Severe pneumonia

On BCPAP

- Check appearance, respiratory efforts, rate, HR, Temp every 8 hourly
- Check SPO2 every 8 hourly

On Low flow O2

- Check appearance, respiratory efforts, rate, HR, Temp every 8 hourly
- Check SPO2 every 8 hourly

On NO O2

- Check appearance, respiratory efforts, rate, HR, Temp every 8 hourly
- Check SPO2 every 8 hourly

- If SPO2 < 85% for more than 60 min, OR
- Granting respiration will not resolved within 60 mins of O2 therapy, or re-appear

- If SPO2 < 85% for more than 60 min, OR
- Granting respiration will not resolved within 60 mins of O2 therapy, or re-appear

- If SPO2 < 90% for more than 5 mins, OR
- Appearance of granting respiration

Treatment Failure

- Immediate measure: Change antibiotics to IV ceftriaxone ± Flucloxacillin
- If not improve, refer to superior facility for further management

- Immediate measure: Change antibiotics to IV ceftriaxone ± Flucloxacillin
- If not improve, refer to superior facility for further management

- Apply BCPAP and follow the 'Follow-up' algorithm for BCPAP

Annex 15 STAKE-HOLDERS INVOLVEMENT

Categories of stakeholders	Who are they in your project(s)?	Analysis: high HP) or low power (LP); high impact on them (HI); low impact on them (LI)	Engagement plan: message, messenger and type of engagement eg 1:1 meeting, invitation to join steering group etc
**1. Consumers: end user of the intervention being tested eg patient, family, service user**	Patient, patients' caregiver, service provider	Integral part of the study and implementation of intervention depends on their availability and proper responses. Without this component evolving of study outcomes may not be possible.	Service providers' message or feedback to caregiver of patient will be continued in different level (different time point) throughout the study period.
**2. Customers: who are we directly trying to impact: clinicians, researchers?**	Clinicians, researchers	Significant role in implementing study activities following the design of the protocol. Administrative, logistic support and scientific guidance will be ensured by them through continued monitoring. Otherwise the actual outcome may be hampered.	Frequent visits of clinicians and researchers in the study hospitals and spend time as much as possible to observe implementation of intervention and gather real life experiences through interaction with relevant personnel. Monthly meeting can be arranged with the participation of researchers and clinicians to discuss together about study progress, findings and other related issues.
**3. Commissioners: payors (individuals, insurers, government)**	Government personnel (e.g., Head in district hospital)	Need to inform about the study to create an amicable environment for implementing the proposed intervention in government hospital facilities, although, they might not have a direct role to implement the study.	Clinicians and researchers will arrange a meeting at the very beginning of the study implementation to create awareness about the study objectives, implementation activities and process. This can help to get a formal permission and their valuable guidance for successful implementation of

			the study. The meeting can be conducted in every six months.
**4. Collaborators: eg NGOs, professional societies, faith organisations, engineers, schools, Rural Unit for Health and Social Affairs…**	icddr,b, Ethiopian Government,<br><br>RESPIRE, University of Edinburg, University of Melbourne	Much important for having continued financial, environmental, logistic and intellectual supports for successful implementation of the intervention.	Study team require informing others about study progress, operational challenges and their potential solutions. Meeting can be held monthly, quarterly, six monthly basis. Continued communication can be maintained through regular Skype call or continued email communication.
**5. Contributors: provide content eg data or previous research**	Published paper in the Lancet 2015, current data of targeted hospital case load from Directorate General of Health Services website of Ethiopia Medical service Directorate at FMOH	Positive results of efficacy trial published in the Lancet influenced to conduct the effectiveness trial and formative research. Data from DGHS website will help to justify the selection of public hospitals for introduction of BCPAP.	Collection of data from DGHS website is required at the very beginning of study implementation.
**6. Channels: to customers and consumers eg TV, radio, clinical magazines and newsletters**	Hospital and research staff (e.g., physicians, nurses, research assistant)	Communication and good relationship among service providers, patient's caregiver and research staff are essential to maintain in delivering the proposed treatment as planned. Hospital physicians and research staff communication with researcher may help to understand the operational	Hospital staff's messages or feedbacks to research staff and research's staff messages to clinicians and researchers will be continued throughout the study period for better documentation of the operational challenges.

		challenges from real life experience. It may not be possible to measure the outcome accurately instead of considering their assistance.	
**7. Commentators: opinion leaders for the customers eg community leaders, press, individuals on social media**	Individuals (e.g., patients caregivers, physicians, nurses, civil surgeon, UH&FPO)	Their opinion may justify the intervention as details as possible from their insights and multiple point of views.	They will express their opinion to the study experts and trained researchers concurrently during conducting interviews and observation.
**8. Champions: eg national policy makers, national offices of agencies eg WHO, World Bank**	Renowned paediatricians', senior physicians, govt decision makers	Their opinion requires creating an enabling policy environment to implement the effectiveness trail within the existing health system. Because, they have influential role in decision making to extend the intervention in other hospitals.	They would be informed by clinicians and researchers about the introduction of bubble CPAP in a meeting before implementing the CPAP. Moreover, they might be invited at mid or end time of study period in dissemination meeting to participate in discussion about key findings of the effectiveness trial, where they can provide valuable feedback and important guidance in adopting the intervention within the existing health system.
**9. Competitors: offer similar or alternative services, undertaking similar research**	Existing treatment protocol for pneumonia patient	Need to measure the outcome of proposed treatment.	The outcome will be measured by the clinicians and researchers at the end of the implementation of proposed intervention.

References

1. UNICEF (2017) Committing to Child Survival: A Promise Renewed. Progress Report 2016
2. Liu L, Oza S, Hogan D, Perin J, Rudan I, et al. (2015) Global, regional, and national causes of child mortality in 2000-13, with projections to inform post-2015 priorities: an updated systematic analysis. Lancet 385: 430-440.
3. You D, Hug L, Ejdemyr S, Idele P, Hogan D, et al. (2015) Global, regional, and national levels and trends in under-5 mortality between 1990 and 2015, with scenario-based projections to 2030: a systematic analysis by the UN Inter-agency Group for Child Mortality Estimation. Lancet.
4. Rahman AE, Moinuddin M, Molla M, Worku A, Hurt L, et al. (2014) Childhood diarrhoeal deaths in seven low- and middle-income countries. Bull World Health Organ 92: 664-671.
5. Duke T, Tamburlini G, Silimperi D, Paediatric Quality Care G (2003) Improving the quality of paediatric care in peripheral hospitals in developing countries. Arch Dis Child 88: 563-565.
6. Graham SM, English M, Hazir T, Enarson P, Duke T (2008) Challenges to improving case management of childhood pneumonia at health facilities in resource-limited settings. Bull World Health Organ 86: 349-355.
7. Duke T, Peel D, Graham S, Howie S, Enarson PM, et al. (2010) Oxygen concentrators: a practical guide for clinicians and technicians in developing countries. Ann Trop Paediatr 30: 87-101.
8. McKiernan C, Chua LC, Visintainer PF, Allen H (2010) High flow nasal cannulae therapy in infants with bronchiolitis. J Pediatr 156: 634-638.
9. Schibler A, Pham TM, Dunster KR, Foster K, Barlow A, et al. (2011) Reduced intubation rates for infants after introduction of high-flow nasal prong oxygen delivery. Intensive Care Med 37: 847-852.
10. Chisti MJ, Salam MA, Smith JH, Ahmed T, Pietroni MA, et al. (2015) Bubble continuous positive airway pressure for children with severe pneumonia and hypoxaemia in Bangladesh: an open, randomised controlled trial. Lancet 386: 1057-1065.
11. Duke T (2014) CPAP: a guide for clinicians in developing countries. Paediatr Int Child Health 34: 3-11.
12. Koti J, Murki S, Gaddam P, Reddy A, Reddy MD (2010) Bubble CPAP for respiratory distress syndrome in preterm infants. Indian Pediatr 47: 139-143.
13. Chisti MJ, Duke T, Ahmed T, Shahunja KM, Shahid ASMSB, et al. (2014) The Use of Bubble CPAP and Humidified High Flow Nasal Cannula Oxygen Therapy in Children with Severe Pneumonia and Hypoxemia: A Systematic Review of the Evidence. Bangladesh Crit Care J 2: 71-78.
14. Liptsen E, Aghai ZH, Pyon KH, Saslow JG, Nakhla T, et al. (2005) Work of breathing during nasal continuous positive airway pressure in preterm infants: a comparison of bubble vs variable-flow devices. J Perinatol 25: 453-458.
15. Courtney SE, Kahn DJ, Singh R, Habib RH (2011) Bubble and ventilator-derived nasal continuous positive airway pressure in premature infants: work of breathing and gas exchange. J Perinatol 31: 44-50.
16. van den Heuvel M, Blencowe H, Mittermayer K, Rylance S, Couperus A, et al. (2011) Introduction of bubble CPAP in a teaching hospital in Malawi. Ann Trop Paediatr 31: 59-65.
17. Buckmaster AG, Arnolda G, Wright IM, Foster JP, Henderson-Smart DJ (2007) Continuous positive airway pressure therapy for infants with respiratory distress in non tertiary care centers: a randomized, controlled trial. Pediatrics 120: 509-518.
18. Kinikar A, Kulkarni R, Valvi C, Gupte N (2011) Use of indigenous bubble CPAP during swine flu pandemic in Pune, India. Indian J Pediatr 78: 1216-1220.

19. Tagare A, Kadam S, Vaidya U, Pandit A, Patole S (2010) A pilot study of comparison of BCPAP vs. VCPAP in preterm infants with early onset respiratory distress. J Trop Pediatr 56: 191-194.
20. Daga S, Mhatre S, Borhade A, Khan D (2014) Home-Made Continuous Positive Airways Pressure Device may Reduce Mortality in Neonates with Respiratory Distress in Low-Resource Setting. J Trop Pediatr 60: 343-347.
21. Koyamaibole L, Kado J, Qovu JD, Colquhoun S, Duke T (2006) An evaluation of bubble-CPAP in a neonatal unit in a developing country: effective respiratory support that can be applied by nurses. J Trop Pediatr 52: 249-253.
22. Yagui AC, Vale LA, Haddad LB, Prado C, Rossi FS, et al. (2011) Bubble CPAP versus CPAP with variable flow in newborns with respiratory distress: a randomized controlled trial. J Pediatr (Rio J) 87: 499-504.
23. Wilson PT, Morris MC, Biagas KV, Otupiri E, Moresky RT (2013) A randomized clinical trial evaluating nasal continuous positive airway pressure for acute respiratory distress in a developing country. J Pediatr 162: 988-992.
24. Chisti MJ, Shahunja KM, Shahid ASMSB, Ahmed T. Outcome of post trial implementation of bubble CPAP in treating childhood severe pneumonia and hypoxemia in Bangladesh; 2018; New Orleans, LA, USA. pp. 649.
25. Walk J, Dinga, P., Banda, C., Msiska, T., Chitsamba, E., Chiwayula, N., Lufesi, N., Mlotha-Mitole, R., Costello, A., Phiri, A., Colbourn, T., McCollum, E. D., Lang, H. J., (2016) Non-invasive ventilation with bubble CPAP is feasible and improves respiratory physiology in hospitalised Malawian children with acute respiratory failure. . Paediatrics & International Child Health 36.
26. Muralidharan Jayashree HK, Sunit Singhi, Karthi Nallasamy, (2016) Use of Nasal Bubble CPAP in Children with Hypoxemic Clinical Pneumonia—Report from a Resource Limited Set-Up. Journal of Tropical Pediatrics 62: 69-74.
27. Laffaye. LFaF (2017) Early Use of Continuos Positive Airway Pressure in the treatment of moderate to severe acute lower respiratory tract infections mong patients younger than 2 years old. Arch Argent Peciatr 115: 274-286.
28. Alice Won DS-R, Arianne L. Baker, Thomas F. Burke and Brett D Nelson. (2018) Bubble CPAP devices for infants and chldren in resource limited settings-a review of the literature. Pediatrics and International Child Health.
29. Wilson PT, Baiden F, Brooks JC, Morris MC, Giessler K, et al. (2017) Continuous positive airway pressure for children with undifferentiated respiratory distress in Ghana: an open-label, cluster, crossover trial. Lancet Glob Health 5: e615-e623.
30. Luo J, Duke T, Chisti MJ, Kepreotes E, Kalinowski V, Li J. (2019) Efficacy of high-flow nasal cannula vs stanadard oxygen therapy or nasal continuous positive airway pressure in children with respiratory distress: a meta-analysis. The Journal of Pediatrics [Epub ahead of print]
31. Anthony ,J Courey R.C (2013). Overview of Mechanical Ventilation in Uptodate 21.2.
32. WHO. Hospital care for children: guidelines for the management of common illnesses with limited resources, 2nd edn. Geneva: World Health Organization, 2013.
