# Supplementary material for: Effectiveness of Bubble Continuous Positive Airway Pressure (BCPAP) for Treatment of Children Aged 1–59 Months with Severe Pneumonia and Hypoxemia in Ethiopia: A Pragmatic Cluster Randomized Controlled Clinical Trial
Source: J Clin Med. 2022 Aug 23;11(17):4934. doi: 10.3390/jcm11174934 (PMC9456562; doi:10.3390/jcm11174934)
Supplement: Supplementary file 1 [file jcm-11-04934-s001.zip › jcm-1817866-supplementary-Appendix SA3.pdf]

<u>**Appendix-SA3**</u>

**Information sheet for Parents/Guardians of children with Severe pneumonia (Stage III)**

**Title of the study*: Feasibility, acceptability, safety, and efficacy/effectiveness of bubble continuous positive airway pressure (CPAP) for treatment of children Aged 1-59 months with severe pneumonia in Ethiopia: a cluster randomized controlled clinical trial***

**Purpose of the research**

Pneumonia is a disease caused by infection in lungs. Pneumonia may become severe and require oxygen therapy along with other medication. There are different forms of delivering oxygen to a child with severe pneumonia. Our aim is to assess the feasibility and effectiveness of locally constructed oxygen delivery system (bubble CPAP) for the treatment of children with severe pneumonia, which is a low cost oxygen delivery method.

**Procedures:** If you volunteer to allow your child to participate in this study and agree with written informed consent, your child will be randomly assigned to oxygen therapy using bubble CPAP or low flow oxygen therapy which is the standard of care. In addition, your child's socio-demographic and clinical data will be filled on questionnaires by the study nurse. If your child doesn't improve while on bubble CPAP or low flow oxygen therapy and needs advanced care, he/she will get the care deemed necessary by the treating physician. Your child's involvement will not affect the care your child is entitled to receive and will not have any influence on the decision of the treating physician.
In case you decide to leave with your child against medical advice during any time of your stay, we will contact you via phone or physically to know the final outcome of your child.

**Risks to study participants:**
**BCPAP**
Although side effects such as nasal trauma, partial nasal obstruction, gastric distension and pneumothorax are reported to occur while using bubble CPAP in neonates, and such complications are found to be extremely rare in older children from studies in Bangladesh, Ghana, Malawi, and India, we do not know its incidence in children with severe pneumonia beyond newborn period who will receive bubble CPAP in Ethiopia. If there is any side effects in Ethiopia, in order to minimize those risks all the necessary precautions will be taken and frequent follow up with routine monitoring of vital signs of your child will be done.

**Low flow (LF)**
This oxygen delivery mechanism is simple and is not expected to result in major complication. But occasionally nasal trauma, partial nasal obstruction and abdominal distension may occur. But all the necessary precautions and follow up will be done to prevent the problems.

**Benefits to study participants:** Your child’s participation is highly beneficial for collecting evidence for further implementation of bubble CPAP for treatment of children with severe pneumonia at a larger scale.

**Outcomes of the study:** The findings of this study will provide relevant information on better delivery modality of oxygen for children with severe pneumonia & hypoxaemia in resource limited settings.

**Right to refuse or withdrawal:** You have full right to refuse or withdraw your child from participating in this study at any time, and if you wish to do so, this will not affect any of the services your child is entitled to receive.

**Confidentiality**: The information of your child related to this study will be kept confidential. Any data related to your child will be kept in a locked cabinet at AHRI and will only be accessed by the investigators. Any identifier related to your child will be removed if the findings of the study are to be published.

**Compensation**: You will not be provided with any compensation by letting your child participate in this study.

**Contact person:** In case of any questions regarding the study or related issues, you can contact any of the following individuals:

**Name of the PI: Dr Meseret Gebre** Tel: 251-0911-977885, Addis Ababa, Ethiopia
**AHRI/ALERT Ethics Review Committee (AAERC), Secretary:** Tel: 251-0118-962183

**Participant’s parent/caregiver consent form (Stage III)**

Date: ___________________________

I have been well informed that MOH & AHRI in collaboration with icddrb would like to carry out a study on “***Feasibility , acceptability, safety, and efficacy/effectiveness of bubble continuous positive airway pressure (CPAP) for treatment of children Aged 1-59 months with severe pneumonia in Ethiopia: a randomized controlled clinical trial*** “.

If I agree on my child’s participation, I am aware that my child will be randomly assigned to oxygen therapy via bubble CPAP or standard low flow oxygen therapy and clinical and demographic data will be collected by the study nurse and that the records will be filled on a questionnaire for the study. I have been also well informed the voluntary nature of participation and that I can withdraw my child withdraw from the study any time. I was informed that my decision won’t affect the care my child is entitled to receive.

I am also informed that if I refuse to let my child participate in the study my child will get oxygen via the standard of care which is low flow oxygen therapy. In addition, I have been given the information that if my child doesn’t improve despite being treated with the locally constructed oxygen delivery system or low flow oxygen therapy and my child needs advanced care he/she will get all the necessary care based on the decision by the treating physician and my child’s involvement in the research won’t affect the decision to be made by the treating physician.

The investigator has also explained to me about the risks associated with bubble CPAP and all the necessary precautions will be taken to avoid possible risks. I have also been informed that In case I decide to leave with my child against medical advice during any time of my stay, I will contacted via phone or physically to know the final outcome of my child.

I have been given enough time to think over allowing the participation of my child in this study before I signed this informed consent. It is therefore with full understanding of the situation that I give my written informed consent for my child to participate in this study.

Name of the parent/care giver: _____________________________Signature _______________

Name of a person taking the consent: _________________________ Signature _________________

Witness: __________________________________Signature ________________

**Amharic version of Information sheet (Stage III)**

በከፍተኛ  ሳንባ ምች ለተጠቁ ልጆች  ወላጅ /አሳዳጊ የቀረበ የመረጃ ቅ💻

የጥናቱ አላማ ፡ልጅዎ በሳንባ ምች ህመም የተጠቃ ሲሆን ብዙ ጊዜ የሚከሰተውም በኢንፌክሽን ምክንያት ነው፡፡ የሳንባ ምች ከፍተኛ ከሆነ ከሌሎች መድሀኒቶች በተጨማሪ ኦክስጅን መስጠት ሊያስፈልግ ይችላል፡፡ ልጅዎ ከሌሎች መድሀኒቶች በተጨማሪ ኦክስጅን ካልተሰጠው በሽታው ተባብሶ ከፍተኛ ደረጃ ሊደርስ ይችላል። በሳንባ ምች ተጠቅቶ/ታ የኦክስጅን እጥረት ላጋጠመው/ማት ህ🌍ን የተለያዩ የኦክስጅን መስጫ መንገዶች ሲኖሩ የአለም ጤና ድርጅት የአፍንጫ ቱቦ ከኦክስጅን ቐት ጋር በማገናኘት እንዲሰጥ ይመክራል። ነገር ግን  በባንግላዲሽ የተደረገ ጥናት እንዳሳየው በሳንባ ምች ለተጠቁና የኦክስጅን እጥረት ለገጠማቸው ህ🌍ናት ኦክስጅን  በበብል ሲፓፕ( ጠቀሜታው የተሻለ ተብሎ ነው የሚታሰብ በቀላሉ የሚገጣጠም መሳሪያ) ቢሰጥ የተሻለ መሆኑን አሳይቶአል።ጥናቱም የተካሄደው በከፍተኛ ክትትልና ተጨማሪ የሰው ሃይል በማካተት ነው። ይህንንም መሳሪያ በሀገራችን ለሚገኙ በሳንባ ምች ለተጠቁ ህጻናት ለመጠቀም እንዲቻል ጥናቱን አነስተኛ የሰው ሀይል ባለባቸው ሆስፒታሎች መስራት አስፈላጊ ነው።ምክንያቱም ይህንን መሳሪያ አነስተኛ የሰው ሀይል እና ክትትል ባለባቸው ሆስፒታሎች ውስጥ ሊሰራ እንደሚችል ማረጋገጥ ያስፈልጋል::: በመሆኑም በዚህ ጥናት ይህንን መሳሪያ በኢትዮጵያ ለሚገኙ በከፍተኛ የሳንባ ምች ለተጠቁ ህ🌍ናት ለማድረስ ሊያጋጥሙ የሚችሉ ተግዳሮቶችን ጥናት እናደርጋለን።

በጥናቱ እንዲሳተፉ የተጠየቁበት ምክንያት

ልጅዎ በከፍተኛ በሳንባ ምች ስለተጠቃና ለዚህም ህመም የተሻለ ህክምና ለማጥናት ይረዱን ዘንድ ነው።

የጥናቱ ሂደት፡- በጥናቱ ውስጥ ልጅዎ እንዲሳተፍ ከፈቀዱና በስምምነት ቅ💻 ላይ ከፈረሙ ልጅዎ አገር ውስጥ በተገጣጠመው የኦክስጅን መስጫ በብል ሲፓፕሲፓፕ( ጠቀሜታው የተሸለ ለው ተብሎ የሚታሰብ በቀላሉ ጠሚገጣም መሳሪያ)  ወይም በተለመደው የኦክስጅን አሰጣጥ ኦክስጅን ይሰጠዋል በተጨማሪም የልጅዎ ማህበራዊ እና ሌሎች የህክምና መረጃዎች ቅ💻 ላይ በጥናቱ ነርስ አማካኝነት ይሞላል።ልጅዎ በዚህ መሳሪያ ወይም በተለመደው የኦክስጅን መስጫ እየታከመ የማይሻለው  እና ከፍተኛ ህክምና የሚያስፈልገው ከሆነ በሀኪሙ አማካኝነት አስፈላጊው ህክምና ይደረግለታል። የልጅዎ በጥናቱ ላይ መሳተፍ ሃኪሙ በሚወስነው ውሳኔ ላይ ምንም አይነት ተ💻ዕኖ አይኖረውም። ምናልባት ልጅዎን ከሃኪም ፈቃድ ውጪ ይዘው ለመሄድ ቢወስኑ የልጅዎን የመጨረሻ ውጤት ለማወቅ አንድ የጥናቱ ቡድን አባል በስልክ አሊያም በአካል ያገኝዎታል።

ስጋትና ጉዳት፡-

**በብል ሲፓፕ፡**

ይህ  የኦክስጅን አሰጣጥ ቀላል የሚባል ሲሆን ከባድ የሚባል ጉዳት ያደርሳል ተብሎ አይጠበቅም።በተጨማሪም በፊት በባንግላዲሽ ጋና እንዲሁም ኡጋንዳ በተደረጉ ጥናቶች ላይ ምንም አይነት ጉዳት አልተከሰተም።ነገር ግን አልፎ አልፎ

የአፍንጫ መቁሰል🔔የአፍንጫ መደፈን የሆድ መወጠርና በሳንባ ሽፋን ውስጥ አየር መሞላት የመሳሰሉት ጉዳት በጨቅላ ህ🖙ናት ላይ ሊከሰቱ እንደሚችሉ የተጠቀሰ ቢሆንም እድሜአቸው ከፍ ባሉ ህ🖙ናት ላይ የመከሰት እድሉ ግን እጅግ በጣም አናሳ ነው

እነዚህንም ጉዳቶች ለመከላከል አስፈላጊው ጥንቃቄ እና ክትትል ይደረጋል

**የተለመደው የኦክስጂን አሰጣጥ(low flow)**

ይህ የኦክስጂን አሰጣጥ ቀላል የሚባል ሲሆን ከባድ የሚባል ጉዳት ያደርሳል ተብሎ አይጠበቅም።ነገር ግን አልፎ አልፎ የአፍንጫ መቁሰል ፣የአፍንጫ በከፊል መደፈን እንዲሁም የሆድ መወጠር ሊከሰት ይችላል፡፤ነገር ግን እነዚህን ጉዳቶች ለመከላከል አስፈላጊው ጥንቃቄ እና ክትትል ይደረጋል

በጥናቱ መሳተፍ ያለው ጠቀሜታ፡-ልጅዎ በዚህ ጥናት መሳተፉ

የዚህ የኦክስጅን አሰጣጥ ዘዴ ጠቀሜታ በጥናት ለማሳየት ተጨማሪ መረጃ ለመሰብሰብና በተመሳሳይ ችግር ለሚጠቁ ህ🖙ናት በሰፊው ለመተግበር የሚያስችል መረጃ ያስገኛል።

<u>የጥናቱ ውጤት፡-</u>ዝቅተኛ ገቢ ባላቸው ሀገሮች ውስጥ የዚህ ጥናት ውጤት በከፍተኛ የሳንባ ምች ለታመሙ ህ🖙ናት የተሻለ የኦክስጅን አሰጣጥን በተመለከተ ጠቃሚ መረጃ ይሰጣል።

<u>በጥናቱ አለመሳተፍ/ፍቃደኛ ያለመሆን፡-</u> በዚህ ጥናት ውስጥ ልጅዎ እንዳይሳተፍ የማድረግ ወይም ከጀመሩ በኋላ የማቋረጥ ሙሉ መብት አልዎት ይህንን ማድረግዎ ልጅዎ ማግኘት የሚገባው/ት ህክምና ላይ ምንም አይነት ተ💻✆ኖ አያመጣም።

የጥናቱ ሚስጥራዊነት<u>፡-ልጅዎን የተመለከተ ማንኛውም መረጃ</u> ሚስጥራዊነቱ የተጠበቀ ነው

<u>የልጅዎ መረጃ በአህሪ በተቆለፈ ሳጥን ውስጥ ይቀመጣል መረጃውን ሊያገኙ የሚችሉት አጥኚዎቹ ብቻ ናቸው ልጅዎን የተመለከተ መረጃ ለህትመት የሚበቃ ከሆነ የልጅዎን ማንነት የሚገልጡ መረጃዎች በሙሉ ይሰረዛሉ</u>

<u>በዚህ ጥናት ልጅዎ በመሳተፉ ምንም አይነት ክፍያ አይኖርም</u>

<u>ስለጥናቱ ጥያቄ ካለዎት የጥናቱን ዋና ተመራማሪ ዶ/ር መሰረት ገብሬ ስ.ቁ 0911977885 ወይም</u>

<u>የአህሪ ኢቲክስ ኮሚቴ ስ.ቁ 0118962183 ማነጋገር ይችላሉ</u>

## Amharic version of consent form (Stage III)

የተሳታፊ ህጻናት ወላጅ/ አሳዳጊ የስምምነት ቅ💻

ቀን---------------------------

የጤና ጥበቃ ድርጅት  እና  አህሪ ከICDDRB ጋር በመተባበር  በብል ሲፓፕ የተባለውን  ኦክስጂን
መስጫ በመጠቀም የሳንባ ምች ያለባቸውን ህ 🖜ናት ማከም

በሚል ርዕስ ላይ ጥናት ለመስራት  እንዳቀዱ ተገልጦልኛል

ልጄ በጥናቱ ላይ እንዲሳተፍ ከተስማማሁ ለልጄ ኦክስጅን የሚሰጠው በብል ሲፓፕ በተባለው መሳሪያ
ወይም በተለመደው የኦክስጅን አሰጣጥ እንደሆነ ተገልጦልኛል እንዲሁም ልጄን የተመለከቱ ማህበራዊና
የጤንነት መረጃዎች በጥናቱ ነርስ አማካይነት እንደሚወሰድ ተነግሮኛል ልጄ በዚህ መሳሪያ ወይም
በተለመደው የኦክስጅን መስጫ እየታከመ የማይሻለው እና ከፍተኛ ህክምና የሚያስፈልገው ከሆነ
በሀኪሙ አማካኝነት አስፈላጊው ህክምና እንደሚደረግለት እንዲሁም  የልጄ በጥናቱ ላይ መሳተፍ
ሃኪሙ በሚወስነው ውሳኔ ላይ ምንም አይነት ተ💻ዕኖ እንደማይኖረው ተገልጾልኛል።

በተጨማሪም በጥናቱ ላይ መሳተፍ በፈቃደ ነት ላይ የተመሰረተ እንደሆነ ተነግሮኛል  ልጄንም
በማንገኛውም ጊዜ ከጥናቱ እንዲያ ቋርጥ ማድረግ እንደምችል ተነግሮኛል የምወስነውም ውሳኔ  ልጄ
በሚያገኘው ህክምና ላይ ምንም ተ💻እኖ እንደማያመጣ ተረድቻለሁ

እንዲሁም በብል ሲፓፕ  ወይም የተለመደው የኦክስጅን አሰጣጥ ሊያመጣው የሚችለውን የጎን
ጉዳትና እንዳይከሰቱም አስፈላጊው ጥንቃቄ እንደሚደረግ ተገልጦልኛል::ምናልባት ልጄን ከሃኪም
ፈቃድ ውጪ ይዠ ለመሄድ ብወስን የልጄን የመጨረሻ ውጤት ለማወቅ አንድ የጥናቱ ቡድን አባል
በስልክ አሊያም በአካል እንደሚያገኘኝ ተነግሮኛል።

በጉዳዩ ላይ ለማሰብ በቂ ጊዜ ከተሰጠኝ  በኋላ በሙሉ መረዳት ልጄ በጥናቱ ላይ እንዲሳተፍ
ፈቃደኝነቴን በፊርማዬ አረጋግጫለሁ

የአሳዳጊ/ወላጅ ስም-------------                    ፊርማ -----------------  ቀን ----
--------------

አሳዳጊው  ከጥናቱ  ተሳታፊ  ጋር  ያለው  ግንኙነት ----------------

ፈቃደኝነት የሚያስፈርመው ስም--------------                    ፊርማ ----------------ቀን-----
-----------

የምስክር ስም--------------                    ፊርማ------------------------ቀን--
--------------
