# Supplementary material for: Effectiveness of Bubble Continuous Positive Airway Pressure (BCPAP) for Treatment of Children Aged 1–59 Months with Severe Pneumonia and Hypoxemia in Ethiopia: A Pragmatic Cluster Randomized Controlled Clinical Trial
Source: J Clin Med. 2022 Aug 23;11(17):4934. doi: 10.3390/jcm11174934 (PMC9456562; doi:10.3390/jcm11174934)

Appendix-SA4

A patient with severe pneumonia and hypoxemia is receiving bubble CPAP oxygen therapy in icddr,b Dhaka hospital

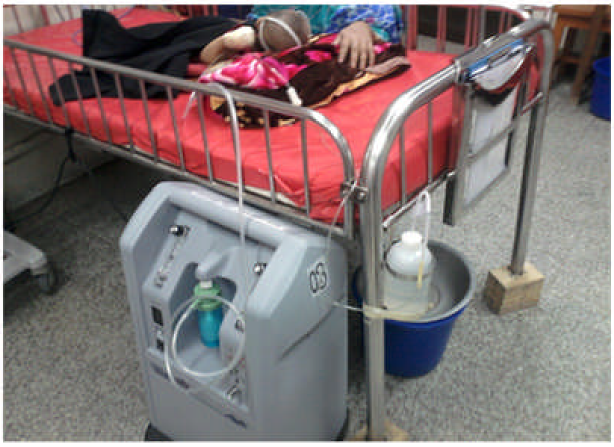

Bubble CPAP at Dhaka Hospital run by oxygen concentrator

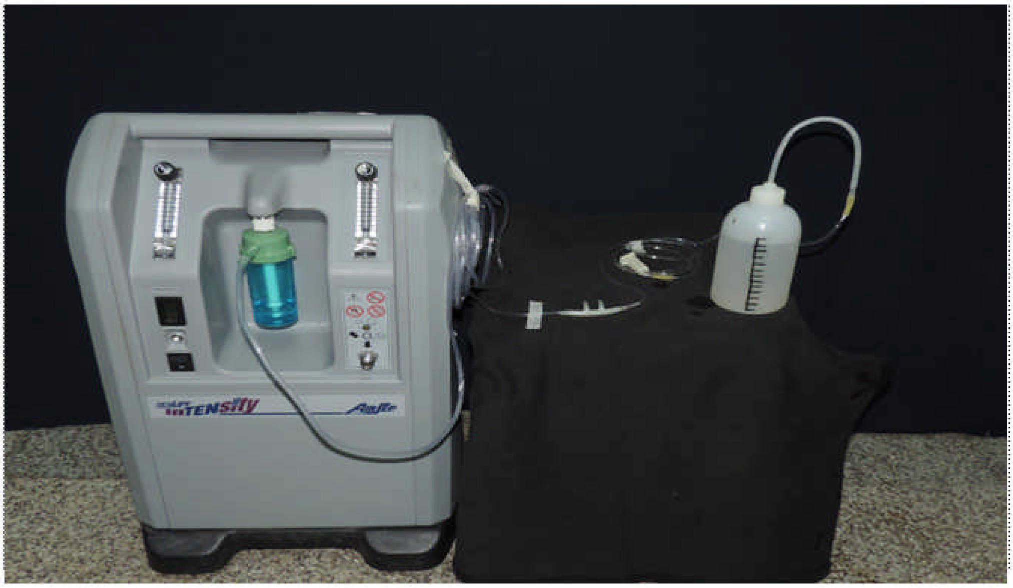

Supplement: Supplementary file 1 [file jcm-11-04934-s001.zip › jcm-1817866-supplementary-Appendix SA4.pdf]
