# Supplementary material for: Effectiveness of Bubble Continuous Positive Airway Pressure (BCPAP) for Treatment of Children Aged 1–59 Months with Severe Pneumonia and Hypoxemia in Ethiopia: A Pragmatic Cluster Randomized Controlled Clinical Trial
Source: J Clin Med. 2022 Aug 23;11(17):4934. doi: 10.3390/jcm11174934 (PMC9456562; doi:10.3390/jcm11174934)
Supplement: Supplementary file 1 [file jcm-11-04934-s001.zip › jcm-1817866-supplementary-Appendix SA5.pdf]

**Appendix SA5: Hospital demographics**

| | Hawassa | Durame | Wolisso | Worabe | Dil Chora | Sabian | Batu | Tulubolo | Fitche | Butajira | Tirunesh Beijing | Shegaw Motta |
|---|---|---|---|---|---|---|---|---|---|---|---|---|
| Catchment population | 1.3 million | 2 million | 600,000 | 2.5-3 million | 2-2.5 million | 1-1.5 million | 745,625 | 650,0001 | 1.5million | 1.5 million | 544,903 | 1.7million |
| Availability of generator | yes | yes | yes | yes | Yes | Yes | yes | yes | yes | yes | yes | yes |
| Oxygen supply | Cylinder, concentrator | Cylinder, concentrator | Cylinder, concentrator | Cylinder, concentrator | Cylinder, concentrator | Cylinder, concentrator | Cylinder, concentrator | Cylinder, concentrator | Cylinder, concentrator | Cylinder, concentrator | Cylinder, concentrator | Cylinder, concentrator |
| Functioning flowmeter | yes | Most | Most | yes | Yes | yes | yes | yes | yes | yes | yes | yes |
| Pulse oximeter | yes | yes | yes | yes | yes | yes | yes | yes | yes | yes | yes | yes |
| ICU/Mechanical ventilator | No | Yes | No | Yes | yes | Yes | Non-functioning | No | Non-functioning | Yes | yes | No |
| CXR | yes | yes | yes | yes | yes | yes | | yes | yes | yes | yes | yes |
| Pneumonia care | Antibiotic oxygen, supportive care | Antibiotic oxygen supportive care | Antibiotic oxygen supportive care | Antibiotic oxygen supportive care | Antibiotic oxygen supportive care | Antibiotic oxygen supportive care | Antibiotic oxygen supportive care | Antibiotic oxygen supportive care | Antibiotic oxygen supportive care | Antibiotic oxygen supportive care | Antibiotic oxygen supportive care | Antibiotic oxygen supportive care |
| Triage | yes | yes | yes | yes | yes | yes | yes | yes | yes | yes | yes | yes |
| Total beds in pediatric ward | 15 | 22 | 65 | 58 | 38 | 17 | 25 | 13 | 16 | 30 | 22 | 16 |
| Total number of nurses in pediatric ward | 13 | 7 | 18 | 15 | 12 | | 3 | 5 | 4 | 9 | 15 | 7 |
| Total number of GPs in pediatric ward | 2 | 1 | 1 | 3 | 2 | 1 | 1 | 1 | 1 | 2 | 2 | 1 |
| Total number of pediatricians in the pediatric ward | 1 | 1 | 1 | 2 | 2 | 1 | 1 | 1 | 1 | 1 | 2 | 1 |
| Total severe pneumonia cases in 12 months | 191 | 237 ( 10 months data) | 476 | 1003 | 126 ( 5 outcomes not recorded) | 106(3 outcomes not recorded) | 250 | 249 | 155 | 1169 | 214 | 714 |

| | | | | | | | | | | | | |
|---|---|---|---|---|---|---|---|---|---|---|---|---|
| Total deaths from severe pneumonia in 12 months | 2 | 4(1 had SAM) | 4 | 9 | 4 | 3 | 6 | 3 | 4 | 2 | 0 | 0 |
| LAMA in 12 months | 4 | 3 | 9 | 5 | 6 | 3 | 4 | 0 | 0 | 3 | 3 | 16 |
| Referred in 12 months | 0 | 4 | 7 | 5 | 0 | 2 | 18 | 0 | 5 | 10 | 18 | 4 |
